# Identification of resident memory CD8^+^ T cells with functional specificity for SARS-CoV-2 in unexposed oropharyngeal lymphoid tissue

**DOI:** 10.1126/sciimmunol.abk0894

**Published:** 2021-10-22

**Authors:** Julia Niessl, Takuya Sekine, Joshua Lange, Viktoria Konya, Marianne Forkel, Jovana Maric, Anna Rao, Luca Mazzurana, Efthymia Kokkinou, Whitney Weigel, Sian Llewellyn-Lacey, Emma B. Hodcroft, Annika C. Karlsson, Johan Fehrm, Joar Sundman, David A. Price, Jenny Mjösberg, Danielle Friberg, Marcus Buggert

**Affiliations:** 1 Department of Medicine, Center for Infectious Medicine, Karolinska Institutet, Stockholm, Sweden.; 2 Division of Infection and Immunity, Cardiff University School of Medicine, University Hospital of Wales, Cardiff, UK.; 3 Biozentrum, University of Basel, Basel, Switzerland.; 4 ISPM, University of Bern, Bern, Switzerland.; 5 Division of Clinical Microbiology, Department of Laboratory Medicine, Karolinska Institutet, Stockholm, Sweden.; 6 Department of Otorhinolaryngology, Karolinska University Hospital, Stockholm, Sweden.; 7 Department of Clinical Science, Intervention and Technology (CLINTEC), Karolinska Institutet, Stockholm, Sweden.; 8 Systems Immunity Research Institute, Cardiff University School of Medicine, University Hospital of Wales, Cardiff, UK.; 9 Department of Surgical Sciences, Otorhinolaryngology and Head and Neck Surgery, Uppsala University, Uppsala, Sweden.

## Abstract

Cross-reactive CD4^+^ T cells that recognize severe acute respiratory syndrome coronavirus 2 (SARS-CoV-2) are more commonly detected in the peripheral blood of unexposed individuals compared with SARS-CoV-2–reactive CD8^+^ T cells. However, large numbers of memory CD8^+^ T cells reside in tissues, feasibly harboring localized SARS-CoV-2–specific immune responses. To test this idea, we performed a comprehensive functional and phenotypic analysis of virus-specific T cells in tonsils, a major lymphoid tissue site in the upper respiratory tract, and matched peripheral blood samples obtained from children and adults before the emergence of COVID-19 (coronavirus disease 2019). We found that SARS-CoV-2–specific memory CD4^+^ T cells could be found at similar frequencies in the tonsils and peripheral blood in unexposed individuals, whereas functional SARS-CoV-2–specific memory CD8^+^ T cells were almost only detectable in the tonsils. Tonsillar SARS-CoV-2–specific memory CD8^+^ T cells displayed a follicular homing and tissue-resident memory phenotype, similar to tonsillar Epstein-Barr virus–specific memory CD8^+^ T cells, but were functionally less potent than other virus-specific memory CD8^+^ T cell responses. The presence of preexisting tissue-resident memory CD8^+^ T cells in unexposed individuals could potentially enable rapid sentinel immune responses against SARS-CoV-2.

## Preexisting T cell immunity to SARS-CoV-2

Preexisting immunity to severe acute respiratory syndrome coronavirus 2 (SARS-CoV-2) infection has been previously identified, because SARS-CoV-2–specific CD4^+^ T cells can be found in the blood of unexposed individuals. However, the presence of preexisting T cells that recognize SARS-CoV-2 in unexposed individuals has not been identified in oropharyngeal tissues. Here, Niessl *et al*. took tonsil samples from children and adults prepandemic and found the presence of SARS-CoV-2–specific CD4^+^ and CD8^+^ T cells. SARS-CoV-2–specific CD8^+^ T cells were more readily detected in the tonsils compared with the blood and displayed a resident memory phenotype but were less functional than CD8^+^ T cells specific for other viruses. Thus, this work suggests that there is tonsil-resident, preexisting T cell memory in some people who have not been exposed to SARS-CoV-2.

## Teaser

Tissue-resident memory CD8^+^ T cells in unexposed oropharyngeal lymphoid tissue exhibit specificity for SARS-CoV-2.

## INTRODUCTION

Coronavirus disease 2019 (COVID-19) has caused millions of deaths worldwide, and hundreds of millions of people have been infected with the causative agent, severe acute respiratory syndrome coronavirus 2 (SARS-CoV-2) (*1*). Large cohort studies have shown that increased age, being male, and various comorbidities, such as obesity, cardiovascular disease, hypertension, and chronic lung diseases, associate with poor outcomes (*2*–*4*). Preexisting immunity against SARS-CoV-2 may also modulate the severity of COVID-19 (*5*).

Several studies have reported the existence of functional T cell responses in the peripheral blood of SARS-CoV-2–unexposed individuals that cross-recognize SARS-CoV-2 (*6*–*14*). Cross-reactive SARS-CoV-2–specific memory (m)CD4^+^ T cells are readily detectable ex vivo in about 20 to 50% of unexposed people and become almost universally detectable after in vitro expansion (*6*), whereas cross-reactive SARS-CoV-2–specific mCD8^+^ T cells are much less common. These preexisting cellular immune responses are likely generated by previous infections with common cold human coronaviruses (HCoVs), which share considerable sequence homology with SARS-CoV-2 (*12*), although other pathogens have also been implicated in this phenomenon (*15*, *16*).

Preexisting T cell immunity associates with negative (*6*) and positive effects on the development of adaptive responses against SARS-CoV-2 (*17*, *18*). However, these studies are limited to analyses of peripheral blood samples, and cross-reactive T cells would likely need to be positioned at sites of viral entry to limit the severity of COVID-19. Immune surveillance is maintained by specialized T cells that recirculate between tissues and the intravascular space via the lymphatic system (*19*), whereas anatomically localized immunity is primarily a function of tissue-resident memory T (T_RM_) cells (*20*), which remain in situ and act as frontline defenders against pathogen invasion (*21*). T_RM_ cells constitutively express CD69 alone or in combination with CD103 and/or CD49a, all of which ensure tissue retention via different mechanisms (*22*–*24*). However, little is known about the function, phenotype, or even the existence of SARS-CoV-2–specific T_RM_ cells in unexposed individuals. This is an important knowledge gap in the light of growing evidence suggesting that T_RM_ cells are critical mediators of protection against viral diseases (*25*–*27*).

In this study, we conducted a flow cytometric survey of virus-specific T cell responses in oropharyngeal (tonsillar) lymphoid tissue and matched peripheral blood samples collected from children and adults before the onset of the current pandemic in 2019. Our results provide previously unknown information regarding the function, phenotype, and tissue compartmentalization of preexisting mCD4^+^ and mCD8^+^ T cells directed against SARS-CoV-2, with potentially important implications for heterologous immune responses.

## RESULTS

### SARS-CoV-2–specific mCD8^+^ T cells preferentially localize to oropharyngeal lymphoid tissue in unexposed individuals

To optimize assays for the detection of virus-specific mCD4^+^ and mCD8^+^ T cell responses in the upper respiratory tract, we first compared the expression of activation-induced markers (AIMs) on the cell surface [programmed death-ligand 1 (PD-L1), CD25, CD134/OX40, and CD137/4-1BB] or the intracellular expression of AIMs (4-1BB and CD154/CD40L) and cytokines [tumor necrosis factor–α (TNF-α) and interferon-γ (IFN-γ)] in the absence or presence of virus peptide stimulation (figs. S1 and S2). AIMs are dynamically regulated among circulating T cells and minimally expressed at baseline (*10*, *28*–*30*). In contrast, we found that AIMs were commonly expressed among tonsillar T cells in the absence of peptide stimulation (fig. S2, A to C), precluding the use of these markers alone for the reliable identification of infrequent antigen-specific responses (fig. S2, D and E), whereas the corresponding background levels of intracellular cytokines were negligible (fig. S2, A to C). We therefore used a combination of intracellular CD40L and TNF-α to identify mCD4^+^ T cell responses and a combination of intracellular 4-1BB and IFN-γ to identify mCD8^+^ T cell responses (fig. S2, D and E).

**Fig. 1.**
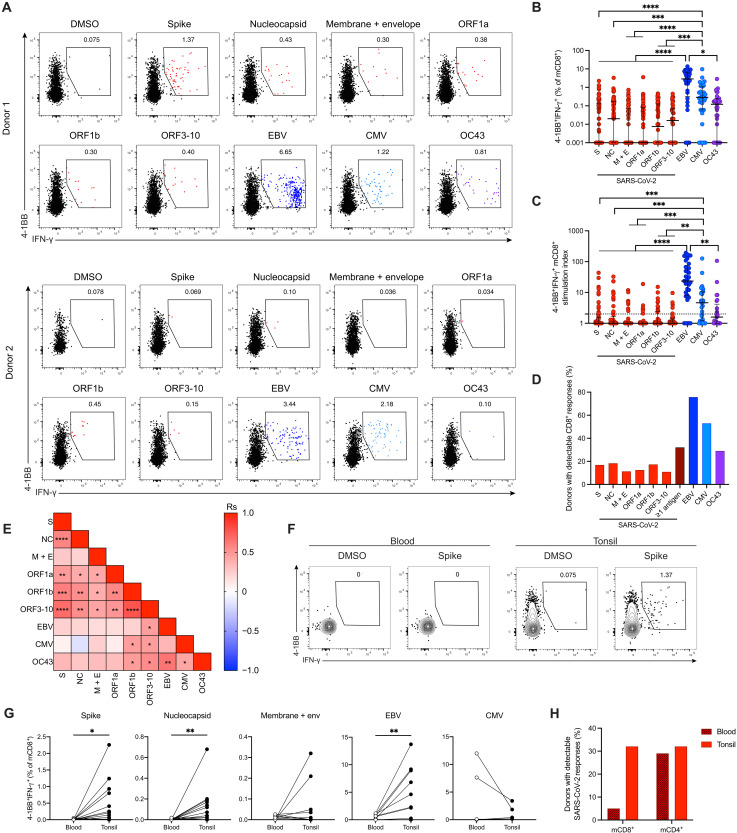
Identification of tonsillar mCD8^+^ T cells specific for SARS-CoV-2 in unexposed individuals. Tonsil cells from children (*n* = 40) and adults (*n* = 41) were stimulated with overlapping peptide pools and analyzed for intracellular expression of 4-1BB/IFN-γ. (**A**) Representative plots showing the gating of 4-1BB^+^IFN-γ^+^ mCD8^+^ T cells from individuals with responses to several SARS-CoV-2 proteins (top) or one SARS-CoV-2 protein (bottom). (**B**) Net frequencies (background subtracted) of 4-1BB^+^IFN-γ^+^ mCD8^+^ T cells. (**C**) Stimulation indices for 4-1BB^+^IFN-γ^+^ mCD8^+^ T cells calculated as fold change relative to the negative control. The dotted line indicates a stimulation index of 2. (**D**) Frequency of tonsil samples with positive antigen-specific mCD8^+^ T cell responses (stimulation index ≥2). (**E**) Correlation matrix showing associations between stimulation indices for virus-specific mCD8^+^ T cells. (**F**) Representative gating of 4-1BB^+^IFN-γ^+^ mCD8^+^ T cells in matched blood and tonsil samples. (**G**) Frequencies of 4-1BB^+^IFN-γ^+^ mCD8^+^ T cells in matched blood and tonsil samples. (**H**) Frequency of blood or tonsil samples with positive mCD4^+^ or mCD8^+^ T cell responses to SARS-CoV-2. (B to E) S, spike; NC, nucleocapsid; and M + E, membrane + envelope. (B and C) Graphs show median ± interquartile range (IQR). Kruskal-Wallis test with Dunn’s posttest. (E) Spearman correlations. (G) Wilcoxon signed-rank test. *P < 0.05; **P < 0.01; ***P < 0.001; ****P < 0.0001.

Tonsil samples were obtained from children (*n* = 40) and adults (*n* = 41) who underwent surgical intervention for obstructive sleep apnea at least 1 year before the emergence of COVID-19 (table S1). Cells were stimulated with overlapping peptide pools spanning the spike, nucleocapsid, membrane, envelope, open reading frame (ORF)1a, ORF1b, and ORF3–10 (ORF3A, ORF6, ORF7A, ORF8, and ORF10) proteins of SARS-CoV-2 and selected immunodominant proteins encoded by Epstein-Barr virus (EBV), cytomegalovirus (CMV), or HCoV-OC43 (Fig. 1A and fig. S3A). Antigen-specific mCD8^+^ T cell responses directed against all four viruses were readily detected in tonsil samples based on the concurrent up-regulation of intracellular 4-1BB and IFN-γ (Fig. 1, A to D). SARS-CoV-2–specific mCD8^+^ T cells occurred at lower frequencies compared with EBV-specific and CMV-specific mCD8^+^ T cells (Fig. 1, B and C) but were nonetheless detected in 26 of the 81 donors (32%) (Fig. 1D). In line with a lack of clear immunodominance patterns in peripheral blood samples (*10*, *11*), these tonsillar mCD8^+^ T cell responses were directed against multiple proteins derived from SARS-CoV-2 (Fig. 1, B to D). Tonsil samples commonly harbored mCD8^+^ T cells specific for EBV (76%) and CMV (53%) but less commonly harbored mCD8^+^ T cells specific for the seasonal HCoV-OC43 (28%) (Fig. 1D). Among tonsil samples with detectable mCD8^+^ T cell responses against SARS-CoV-2, 50% showed reactivity against a single antigen, and <25% showed reactivity against more than two antigens (Fig. 1A and fig. S3B). Stimulation indices were correlated positively among different SARS-CoV-2 antigens, and ORF1b-specific and/or ORF3–10-specific responses correlated positively with those directed against EBV, CMV, and HCoV-OC43 (Fig. 1E). The latter also demonstrated a positive correlation with responses directed against EBV and CMV (Fig. 1E).

Tonsillar mCD4^+^ T cell responses directed against SARS-CoV-2 were detected in a subset of individuals based on the concurrent up-regulation of intracellular CD40L and TNF-α (fig. S4, A to D). Higher background expression of these markers was observed among tonsillar mCD4^+^ T cells compared with tonsillar mCD8^+^ T cells in some individuals, likely reflecting greater levels of basal activation and effectively raising the detection threshold for antigen-specific responses (Fig. 1A and fig. S4A). Despite these limitations, we detected SARS-CoV-2–reactive mCD4^+^ T cell responses in 32% of tonsil samples (fig. S4E), and in total, we detected SARS-CoV-2–reactive mCD4^+^ and/or mCD8^+^ T cell responses in 49% of tonsil samples (fig. S4F).

Cross-reactive SARS-CoV-2–specific mCD4^+^ T cell responses appear to be more common in peripheral blood than cross-reactive SARS-CoV-2–specific mCD8^+^ T cell responses (*6*–*11*). Our analysis suggested that SARS-CoV-2–specific mCD4^+^ and mCD8^+^ T cells were present at similar frequencies in SARS-CoV-2–unexposed oropharyngeal lymphoid tissue (fig. S4F). To address this potential discrepancy, we compared virus-specific mCD4^+^ and mCD8^+^ T cell responses in matched tonsil and peripheral blood samples from a subset of donors in our cohort. SARS-CoV-2–specific mCD4^+^ T cells were detected at similar frequencies in both compartments (fig. S4G), whereas SARS-CoV-2–specific mCD8^+^ T cells were detected almost exclusively in the tonsils (Figs. 1, F to H). EBV-specific mCD8^+^ T cells were also found predominantly in the tonsils (Fig. 1G), whereas CMV-specific T cells were mainly found in the circulation (Fig. 1G), as described previously (*31*–*33*). This approach enabled the sensitive detection of virus-specific CD4^+^ and CD8^+^ T cell responses, allowing us to show that SARS-CoV-2–reactive mCD8^+^ T cells were preferentially localized to the tonsils in unexposed individuals.

### SARS-CoV-2–specific mCD8^+^ T cells occur at similar frequencies and prevalence rates in oropharyngeal lymphoid tissue from unexposed children and adults

**Fig. 2.**
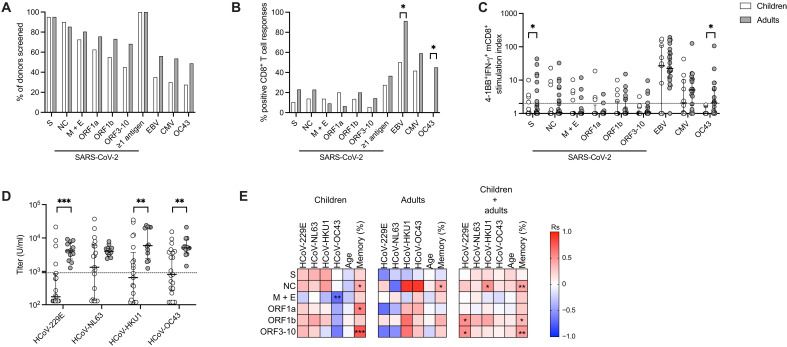
Magnitude and prevalence of tonsillar mCD8^+^ T cells specific for SARS-CoV-2 in unexposed children and adults. Virus-specific tonsillar mCD8^+^ T cell responses were compared between children (white bars/symbols; *n* = 40) and adults (gray bars/symbols; *n* = 41). (**A**) Overall frequency of tonsil samples tested with individual peptide pools. (**B**) Overall frequency of tonsil samples with positive mCD8^+^ T cell responses (stimulation index ≥2) to different antigens. (**C**) Stimulation indices for 4-1BB^+^IFN-γ^+^ mCD8^+^ T cells calculated as fold change relative to the negative control. The dotted line indicates a stimulation index of 2. (**D**) Antibody titers (units per milliliter) for HCoVs in children (*n* = 21) and adults (*n* = 12). The dotted line represents a seropositivity threshold calculated based on SARS-CoV-2 spike trimer-specific antibody titers as shown in fig. S5A. (**E**) Correlation matrices showing associations between stimulation indices for SARS-CoV-2–specific mCD8^+^ T cell responses, HCoV antibody titers, age, and total frequencies of mCD8^+^ T cells for children, adults, or both cohorts. (B) Fisher’s exact test. (C and D) Graphs show median ± IQR. Mann-Whitney test. (E) Spearman correlation. *P < 0.05; **P < 0.01; ***P < 0.001.

Preexisting immunity may explain why children are less susceptible to severe COVID-19 (*34*). We therefore evaluated virus-specific tonsillar mCD8^+^ T cell responses in young children (age 2 to 5 years) and adults (age 28 to 67 years) (Fig. 2A). SARS-CoV-2–specific mCD8^+^ T cells were distributed similarly between groups in terms of frequency and overall prevalence, with the exception of responses directed against the spike protein, which occurred at lower magnitudes in children (Fig. 2, B and C). EBV-specific mCD8^+^ T cells were less common in children (Fig. 2B), likely reflecting lower seroprevalence rates (*35*). Similarly, HCoV-OC43–specific mCD8^+^ T cells were undetectable in tonsil samples from children (Fig. 2B), consistent with lower seroprevalence rates for HCoVs-OC43 and other HCoVs (Fig. 2D, fig. S5A, and table S2). HCoV-OC43–specific responses were also undetectable in some HCoV-OC43–seropositive adults (fig. S5B), confirming the previously reported discordance between humoral and cellular immunity against HCoVs (*36*).

The presence of EBV-specific mCD8^+^ T cell responses associated with the occurrence of SARS-CoV-2–specific mCD8^+^ T cell responses in children but not in adults (table S3). In addition, the stimulation indices for mCD8^+^ T cell responses directed against the SARS-CoV-2 nucleocapsid, ORF1b, and ORF3–10 proteins correlated positively with certain HCoV-specific antibody titers (Fig. 2E and fig. S5C), and SARS-CoV-2–specific mCD8^+^ T cell responses were identified only in children with detectable antibodies specific for HCoV-HKU-1 or HCoV-NL63 and not in children with detectable antibodies specific for HCoV-229E or HCoV-OC43 (fig. S5D). No significant correlations were detected between age and SARS-CoV-2–derived antigen-specific mCD8^+^ T cell response stimulation indices in children or adults (Fig. 2D). However, positive correlations were detected between mCD8^+^ T cell responses directed against nucleocapsid, ORF1a, and ORF3–10 in children, and nucleocapsid only in adults, and the overall size of the mCD8^+^ T cell pool (Fig. 2E and fig. S5C). Accordingly, previous exposure to other viruses, including HCoVs, not age, appeared to drive the formation of oropharyngeal mCD8^+^ T cell responses against SARS-CoV-2.

### SARS-CoV-2–specific mCD8^+^ T cells exhibit a follicular homing and T_RM_ phenotype in oropharyngeal lymphoid tissue from unexposed individuals

To extend these findings, we investigated the phenotypic characteristics of virus-specific mCD8^+^ T cells in the tonsils, with a particular focus on markers of follicular homing and tissue residency. The expression of surface markers in this analysis, including CD69, was not influenced by stimulation in these experiments (fig. S6, A to C). mCD8^+^ T cells specific for different SARS-CoV-2–derived antigens were pooled for downstream analyses on the grounds of phenotypic homogeneity (fig. S6, D and E).

**Fig. 3.**
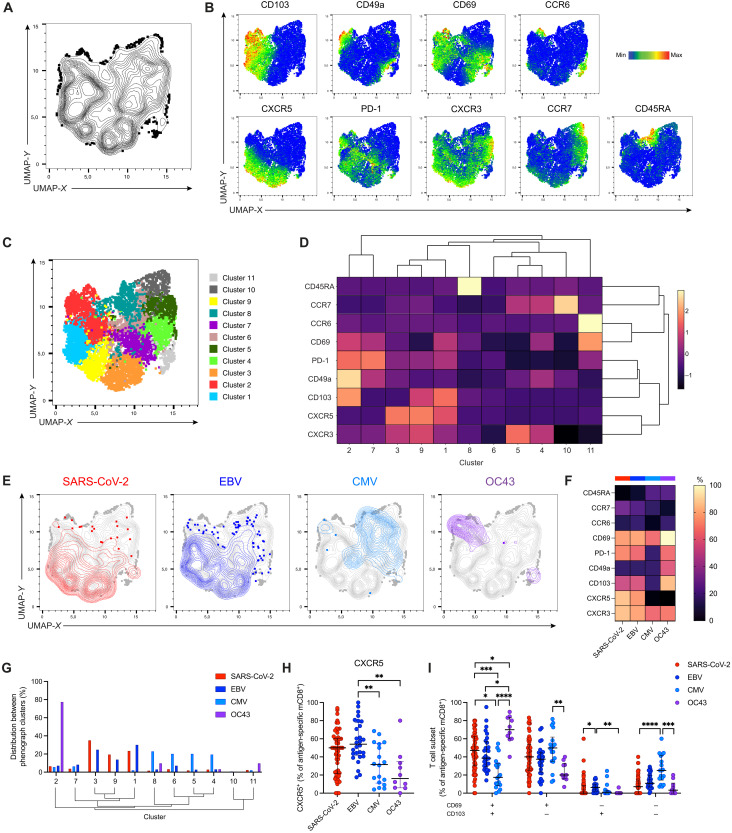
Tonsillar SARS-CoV-2–specific mCD8^+^ T cells in unexposed individuals express CD103, CD69, and CXCR5. (**A**) UMAP representation showing the clustering of concatenated total and virus-specific mCD8^+^ T cells from representative individuals (*n* = 4). (**B**) Expression of individual surface markers overlaid on the UMAP plot derived from (A). (**C**) UMAP representation derived from (A) with subpopulations (*n* = 11) identified using Phenograph. (**D**) Hierarchical clustering of expression intensity for the indicated markers in each subpopulation identified in (C). (**E**) Distribution of virus-specific mCD8^+^ T cells on the UMAP plot derived in (A). (**F**) Median percent expression of the indicated markers among virus-specific mCD8^+^ T cells. (**G**) Distribution of virus-specific mCD8^+^ T cells among clusters derived using Phenograph. Clusters are hierarchical as indicated in (D). (**H**) Frequency of CXCR5^+^ virus-specific mCD8^+^ T cells. (**I**) Comparison of virus-specific mCD8^+^ subsets based on the expression of CD69/CD103. (H and I) Graphs show median ± IQR. Kruskal-Wallis test with Dunn’s posttest. *P < 0.05; **P < 0.01; ***P < 0.001; ****P < 0.0001.

Total and virus-specific mCD8^+^ T cells were concatenated from four representative individuals, and nonlinear relationships among individual cells were assessed using Uniform Manifold Approximation and Projection (UMAP) (Fig. 3A). Distinct topographical regions were delineated by overlaying the expression of analyzed phenotypic markers, namely, CD103, CD49a, CD69, CCR6, CXCR5, PD-1, CXCR3, CCR7, and CD45RA (Fig. 3B). Phenograph analysis revealed 11 unique clusters in the UMAP space (Fig. 3C), each with a distinct pattern of surface marker expression (Fig. 3D). Overlays of antigen-specific mCD8^+^ T cells revealed unique clustering based on specificity (Fig. 3E). SARS-CoV-2–specific and EBV-specific mCD8^+^ T cells were found mainly in clusters 1, 3, and 9 (Fig. 3G), which displayed high expression of the T_RM_ markers CD103 and CD69 and the follicular homing marker CXCR5 (Fig. 3, B and D). In contrast, CMV-specific mCD8^+^ T cells were distributed among clusters 4 to 8, which included populations expressing high levels of CD45RA and populations lacking CD103 and CXCR5 (Fig. 3, B to G). HCoV-OC43–specific mCD8^+^ T cells were found predominantly in cluster 2, which displayed high expression levels of the T_RM_ markers CD103, CD49a, and CD69. Manual gating analysis further confirmed these phenotypic differences among specificities with respect to CD103, CD69, and CXCR5 (Fig. 3, H and I, and fig. S6, F and G). SARS-CoV-2–specific and EBV-specific tonsillar mCD8^+^ T cells were therefore characterized by a follicular homing and T_RM_ phenotype, which was similar in children and adults (fig. S6, H and I).

### Functional properties of virus-specific mCD8^+^ T cells in oropharyngeal lymphoid tissue

**Fig. 4.**
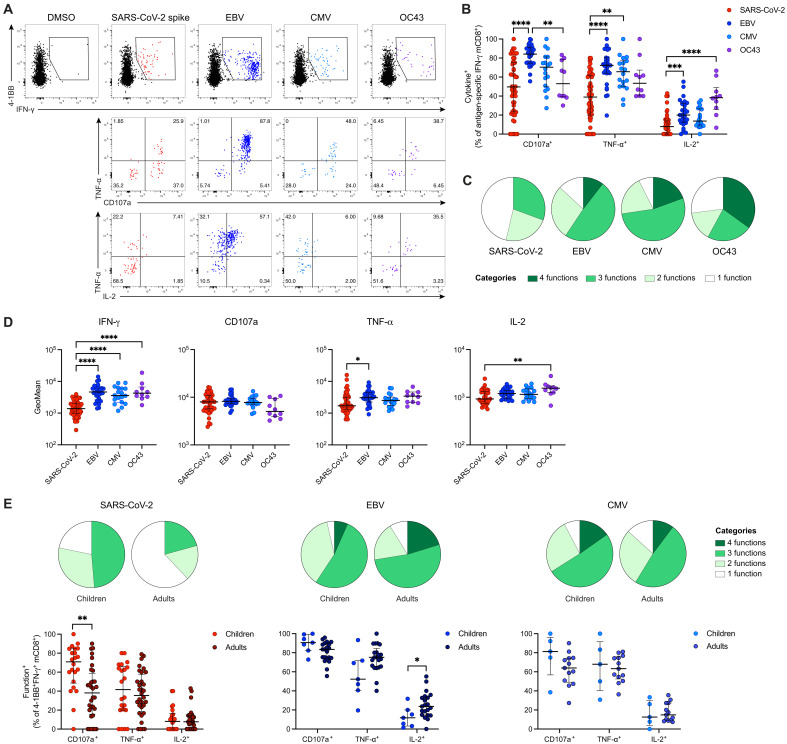
Functional profiles of virus-specific tonsillar mCD8^+^ T cells. (**A**) Representative plots showing coexpression of CD107a/TNF-α or IL-2/TNF-α among 4-1BB^+^IFN-γ^+^ virus–specific mCD8^+^ T cells. (**B**) Frequencies of CD107a^+^, TNF-α^+^, or IL-2^+^ virus–specific mCD8^+^ T cells. (**C**) Pie chart summarizing the functional profiles of virus-specific mCD8^+^ T cells. (**D**) Geometric mean (GeoMean) fluorescence values for IFN-γ, CD107a, TNF-α, and IL-2 among function^+^ virus-specific mCD8^+^ T cells. (**E**) Functional profiles and frequencies of CD107a^+^, TNF-α^+^, or IL-2^+^ virus–specific mCD8^+^ T cells in children and adults. Graphs show median (C and E) or median ± IQR (B, D, and E). (B and D) Kruskal-Wallis test with Dunn’s posttest. (E) Mann-Whitney test. *P < 0.05; **P < 0.01; ***P < 0.001; ****P < 0.0001

To assess the functionality of virus-specific tonsillar mCD8^+^ T cells, we quantified the surface mobilization of CD107a and the intracellular expression of TNF-α and interleukin-2 (IL-2) alongside 4-1BB and IFN-γ in response to peptide stimulation (Fig. 4A). Preexisting mCD8^+^ T cells specific for SARS-CoV-2 mobilized CD107a and up-regulated TNF-α and IL-2 as individual functions less commonly than mCD8^+^ T cells specific for EBV, CMV, or HCoV-OC43 (Fig. 4B). In addition, mCD8^+^ T cells specific for EBV, CMV, or HCoV-OC43 were highly polyfunctional and frequently expressed IFN-γ together with CD107a, TNF-α, and/or IL-2 (Fig. 4C and fig. S7A), whereas mCD8^+^ T cells specific for SARS-CoV-2 were generally oligofunctional (Fig. 4C and fig. S7A) and expressed comparatively lower amounts of IFN-γ, TNF-α, and IL-2 on a per-cell basis (Fig. 4D). A similar pattern of suboptimal functionality has been described previously for cross-reactive intravascular SARS-CoV-2–specific mCD4^+^ T cells, likely reflecting low-avidity recognition of the corresponding nonprimary antigens (*6*).

mCD8^+^ T cells specific for each virus mobilized CD107a to a similar extent and produced largely equivalent amounts of IFN-γ, TNF-α, and IL-2 on a per-cell basis in children and adults (fig. S7B). The functional profiles of EBV-specific and CMV-specific mCD8^+^ T cells were also similar in each group, although EBV-specific mCD8^+^ T cells more commonly produced IL-2 in adults (Fig. 4E). In contrast, SARS-CoV-2–specific mCD8^+^ T cells more commonly mobilized CD107a and more commonly expressed other functional markers alongside IFN-γ in children (Fig. 4E and fig. S7C). Together, these data suggest that SARS-CoV-2–unexposed children harbored higher frequencies of polyfunctional SARS-CoV-2–reactive mCD8^+^ T cells in tonsillar lymphoid tissue compared with unexposed adults.

## DISCUSSION

CD8^+^ T cells are essential for immune control and clearance of previously encountered viruses. CD8^+^ T cells may also provide a degree of heterologous protection against previously unencountered viruses expressing structurally related or unrelated antigens that elicit functional responses within the preexisting memory pool as a consequence of cross-reactivity (*37*–*39*). In line with the concept of heterologous immunity, previous studies have demonstrated a high prevalence of SARS-CoV-2–specific mCD4^+^ T cells and a relative paucity of SARS-CoV-2–specific mCD8^+^ T cells in the peripheral blood of SARS-CoV-2–unexposed individuals (*8*–*14*). However, it has remained unclear to what extent such preexisting T cell responses against SARS-CoV-2 exist in tissues, which is an important omission, especially given the critical role of anatomically localized immunity in other viral infections (*25*–*27*). The present study was designed to address this knowledge gap and provide a comprehensive picture of preexisting SARS-CoV-2–specific mCD4^+^ and mCD8^+^ T cell immunity in the tonsils, representing key lymphoid organs in the upper respiratory tract, and the intravascular circulation. We found that SARS-CoV-2–specific mCD4^+^ T cells were distributed evenly between these compartments, whereas SARS-CoV-2–specific mCD8^+^ T cells were almost exclusively present in the tonsils. Earlier studies confined to analyses of peripheral blood samples have therefore likely underestimated the overall prevalence of heterologous mCD8^+^ T cell responses against SARS-CoV-2.

Tonsils play an important role in immune defense against inhaled or orally acquired pathogens, highlighted by the increased risk of upper respiratory tract infections in children after tonsillectomy (*40*). Although much emphasis has been placed on the nasal/respiratory route of viral transmission (*41*), the oral cavity, including the tonsils, can also be infected and potentially acts as a primary site for the acquisition of SARS-CoV-2 (*42*). We found that SARS-CoV-2–specific mCD8^+^ T cells expressed markers of tissue residency, potentially forming a sentinel immune response at a key site of viral entry (*27*). T_RM_ cells function partly as innate-like sensors (*43*) and may therefore trigger an early type I IFN response, which commonly associates with nonsevere forms of COVID-19. An early CD8^+^ T cell proliferation signature coinciding with a type I IFN response has been detected in asymptomatic and mild cases of COVID-19 before the detection of SARS-CoV-2 (*44*). This phenomenon associates with accelerated viral clearance and a less severe cytokine storm at the site of infection, as demonstrated for cross-reactive T_RM_ cell responses directed against influenza virus, SARS-CoV-1, or Middle East respiratory syndrome–related coronavirus in mice (*37*, *45*). It remains unclear whether such early activation of SARS-CoV-2–specific CD8^+^ T cells reflects preexisting memory recall or rapid de novo priming from the naive pool in milder forms of COVID-19. However, recent infection with HCoVs associates with less severe disease and lower mortality rates after subsequent infection with SARS-CoV-2 (*17*). In addition, preexisting virus-specific mCD4^+^ and mCD8^+^ T cells numerically expand in the peripheral blood after infection with SARS-CoV-2 (*46*, *47*), and more robust cellular and humoral immune responses occur after infection in patients with preexisting CD4^+^ T cell responses directed against SARS-CoV-2 (*18*). Further studies incorporating larger numbers of patients, matched tissue samples, and longitudinal analyses will nonetheless be required to understand the full impact of preexisting SARS-CoV-2–specific mCD4^+^ and mCD8^+^ T cell responses on the outcome of COVID-19.

EBV-specific mCD8^+^ T cells preferentially localized to the tonsils, where they exhibited a follicular homing and T_RM_ phenotype akin to SARS-CoV-2–specific mCD8^+^ T cells, whereas CMV-specific mCD8^+^ T cells, which mostly lacked T_RM_ markers, are largely confined to the intravascular circulation (*33*). This anatomical dichotomy makes sense biologically, given the distinct tropism patterns of EBV and CMV with EBV being transmitted orally and mainly infecting B lymphocytes residing in lymphoid tissues and epithelial cells and CMV spreading throughout the body to infect multiple cell types and organs. CD69^+^CD103^+^ tonsillar mCD8^+^ T cells are located near the epithelial barrier surface, likely facilitated by high expression levels of E-cadherin (*33*). Accordingly, the preexisting SARS-CoV-2–specific tonsillar mCD8^+^ T cells identified here might have been positioned near sites of viral entry, potentially enabling them to spearhead an early immune response.

Children and young adults are less prone to severe COVID-19 than older adults (*48*). Although there is a decline in cross-reactive SARS-CoV-2–specific humoral and cellular immunity with age (*49*, *50*), we found that preexisting SARS-CoV-2–specific tonsillar mCD8^+^ T cells in children and adults were similar in terms of frequency, phenotype, and overall prevalence. Note that we did not detect HCoV-OC43–specific mCD8^+^ T cell responses in tonsils obtained from children, despite the fact that some of these individuals were HCoV-OC43 seropositive and/or harbored detectable mCD4^+^ T cell responses directed against HCoV-OC43. This discrepancy could reflect sensitivity limitations in our approach to the detection of antigen-specific mCD8^+^ T cells directly ex vivo. In addition, HCoV-specific mCD8^+^ T cell responses may be liable to decay (*51*), and repeated viral exposure may be necessary to induce long-lived HCoV-specific CD8^+^ T_RM_ cells (*52*). A similar discrepancy is reported in a study of peripheral blood samples comparing cellular and humoral responses against HCoVs (*36*).

It is likely that preexisting SARS-CoV-2–specific mCD4^+^ and mCD8^+^ T cell immunity is generated via previous encounters with HCoVs. In line with this notion, we found that SARS-CoV-2–specific mCD8^+^ T cell response frequencies correlated with certain HCoV-specific antibody titers, and in children, we identified SARS-CoV-2–specific mCD8^+^ T cell responses almost exclusively in individuals with detectable antibodies specific for HCoV–HKU-1 or HCoV-NL63. However, we also found that the presence of EBV-specific mCD8^+^ T cell responses in children, likely reflecting persistent infection, associated with the occurrence of SARS-CoV-2–specific mCD8^+^ T cell responses potentially arising as a consequence of heterologous immunity (*38*). Alternatively, the presence of EBV-specific mCD8^+^ T cell responses could identify children with greater overall levels of exposure to endemic pathogens. Consistent with either possibility, the stimulation indices for some SARS-CoV-2–specific mCD8^+^ T cell responses correlated with the overall size of the mCD8^+^ T cell pool, which provides a general measure of immunological experience (*6*). Accordingly, prior antigen experience is likely the key driver of heterologous immunity, irrespective of the precise mechanism, shaping a diverse repertoire of mCD8^+^ T cells incorporating various specificities with the ability to cross-recognize SARS-CoV-2.

Overall, we detected SARS-CoV-2–specific mCD4^+^ T cell responses in 29% of unexposed peripheral blood samples, which is within the range reported previously (*8*–*14*). However, the true prevalence may be higher, because recovered cell numbers in some cases precluded a full assessment of all viral antigen specificities. A similar caveat applies to the identification of SARS-CoV-2–specific mCD8^+^ T cell responses in peripheral blood samples and the identification of SARS-CoV-2–specific mCD4^+^ and mCD8^+^ T cell responses in the tonsils. In addition, our study was limited by detection based on the production of IFN-γ or TNF-α, which excluded cells that did not produce these cytokines in response to antigen encounter. Furthermore, a high level of background activation without any peptide stimulation, in some individuals, may prevent the detection of low-frequency virus-specific T cell responses. It should also be noted that our findings cannot be taken in isolation as evidence of heterologous immune protection against SARS-CoV-2.

In summary, we have shown that SARS-CoV-2–unexposed children and adults commonly harbor tonsillar CD8^+^ T_RM_ cells that react with SARS-CoV-2. Additional studies are now warranted to determine whether these preexisting CD8^+^ T_RM_ cells lead to early viral containment, potentially mitigating the course of COVID-19.

## MATERIALS AND METHODS

### Study design

The objective of this study was to characterize SARS-CoV-2–reactive mCD4^+^ and mCD8^+^ T cell responses in tonsillar lymphoid tissue and matched peripheral blood samples obtained from children and adults before the current pandemic between 2015 and 2018. Functional and phenotypic analyses were extended for comparative purposes to include mCD4^+^ and mCD8^+^ T cell responses elicited by natural infections with EBV, CMV, and HCoV-OC43.

### Human subjects and ethics

Tonsil samples and matched peripheral blood mononuclear cells (PBMCs) were obtained from individuals undergoing tonsillectomy for obstructive sleep apnea at the Department of Otorhinolaryngology, Karolinska University Hospital, Stockholm, Sweden. All samples were collected between 2015 and 2018. The study was approved by the regional ethics committee in Stockholm (2014/1000-31 for the child cohort and 2015/755-31, 2016/128-32, and 2017/2275-32 for the adult cohort). Written informed consent was obtained from all participants or their legal representatives in accordance with the Declaration of Helsinki. Whole tonsils were cut into small pieces, ground through a cell strainer (pore size = 100 μm), filtered through another cell strainer (pore size = 40 μm), and resuspended in phosphate-buffered saline (PBS). Mononuclear cells were isolated via standard density gradient centrifugation and cryopreserved in fetal bovine serum (FBS) containing 10% dimethyl sulfoxide (DMSO). PBMCs were isolated and cryopreserved similarly. Plasma samples were collected from some individuals (*n* = 21 children and *n* = 12 adults) and stored at −80°C. The adult tonsil cohort was dominated by male participants in this study, because obstructive sleep apnea is more common in adult men than in adult women (*53*). Plasma samples were also collected from two convalescent individuals recovering from infection with SARS-CoV-2 (ethical approval: dnr 2019-05757 and dnr 2014/1000-31/1) and stored at −80°C. Donor characteristics are summarized in table S1.

### Peptides

Lyophilized pools of overlapping peptides spanning the relevant proteins were reconstituted in DMSO and further diluted to 50 or 100 μg/ml in PBS. Reconstituted pools were aliquoted and stored at −20°C. Overlapping peptide pools spanning the SARS-CoV-2 spike (Prot_S) and nucleocapsid (Prot_N) proteins were obtained from Miltenyi Biotec. Overlapping peptide pools spanning the SARS-CoV-2 membrane and envelope (membrane + env) proteins and the ORF3–10 proteins were synthesized using sequences derived from GenBank reference MT093571.1 (Sigma-Aldrich). Overlapping peptide pools spanning the SARS-CoV-2 ORF1a and ORF1b proteins were generated by combining non-structural protein (NSP)1 to 11 and NSP12 to 16, respectively (JPT Peptide Technologies). Overlapping peptide pools spanning the EBV BZLF1, EBV nuclear antigen (EBNA)-1, EBNA-3a, and latent membrane protein (LMP)2 proteins were purchased from JPT Peptide Technologies. Overlapping peptide pools spanning the CMV pp65 and IE-1 proteins were purchased from Peptides & Elephants. Metadata for all available HCoV-OC43 sequences on the Virus Pathogen Database and Analysis Resource (ViPR) website were downloaded on 27 January 2020. Sequences were filtered to include isolates from humans and exclude isolates from cell passage or vaccine development studies. One sequence per country per year was selected to create a representative set of HCoV-OC43 sequences. The spike, nucleocapsid, and membrane gene amino acid translations (as available) were downloaded from GenBank. The amino acid sequences for each gene were then translated using the “--auto” setting in MAFFT (*54*), and a consensus sequence for each gene was generated using the “dump_consensus()” method with default settings in Biopython (*55*). Overlapping peptide pools spanning these HCoV-OC43 spike, nucleocapsid, and membrane (OC43) protein consensus sequences were synthesized by Sigma-Aldrich. The relevant peptides were combined to generate single pools for EBV, CMV, and HCoV-OC43.

### Intracellular cytokine staining

Cryopreserved tonsil cells and PBMCs were thawed quickly, resuspended in RPMI 1640 containing 10% FBS, 1% l-glutamine, and 1% penicillin/streptomycin (complete medium) in the presence of deoxyribonuclease (DNase) I (10 U/ml; Sigma-Aldrich), and rested at 1 × 10^6^ to 2 × 10^6^ cells per well in 96-well U-bottom plates (Corning) for 5 hours at 37°C. The medium was then supplemented with anti–CXCR5-BB515 (clone RF8B2; BD Biosciences) followed 15 min later by the relevant peptide pools (0.5 μg/ml per peptide) and a further 1 hour later by brefeldin A (1 μg/ml; Sigma-Aldrich), monensin (0.7 μg/ml; BD Biosciences), and anti–CD107a-BV785 (clone H4A3; BioLegend). In one experiment, tonsil cells were stained with anti–CD69-BUV563 (clone FN50; BD Biosciences) for 30 min at room temperature and washed in complete medium before stimulation (fig. S6A). Negative control wells contained equivalent DMSO, and positive control wells contained staphylococcal enterotoxin B (SEB; 0.5 μg/ml; Sigma-Aldrich). After 9 hours, cells were washed in PBS supplemented with 2% FBS and 2 mM EDTA [fluorescence-activated cell sorting (FACS) buffer] and stained with anti–CCR4-BB700 (clone 1G1; BD Biosciences), anti–CCR6-BUV737 (clone 11A9; BD Biosciences), anti–CCR7–APC-Cy7 (clone G043H7; BioLegend), and anti–CXCR3-BV750 (clone 1C6; BD Biosciences) for 10 min at 37°C. Additional surface stains were performed for 30 min at room temperature in the presence of Brilliant Stain Buffer Plus (BD Biosciences). Viable cells were identified by exclusion using a LIVE/DEAD Fixable Aqua Dead Cell Stain Kit (Thermo Fisher Scientific). Cells were then washed in FACS buffer and fixed/permeabilized using a FoxP3 transcription factor staining buffer set (Thermo Fisher Scientific). Intracellular stains were performed for 30 min at room temperature. Stained cells were washed in FACS buffer, fixed in PBS containing 1% paraformaldehyde (PFA; Biotium), and acquired using a FACSymphony A5 (BD Biosciences). Flow cytometry reagents are listed in table S4.

### Surface staining for AIMs

Cryopreserved tonsil cells and PBMCs were thawed quickly, resuspended in complete medium in the presence of DNase I (10 U/ml; Sigma-Aldrich), and rested at 1 × 10^6^ to 2 × 10^6^ cells per well in 96-well U-bottom plates (Corning) for 5 hours at 37°C. The medium was then supplemented with the relevant peptide pools (0.5 μg/ml per peptide). Negative control wells contained equivalent DMSO, and positive control wells contained SEB (0.5 μg/ml; Sigma-Aldrich). After 18 hours, cells were washed in FACS buffer and stained with anti–CCR7–APC-Cy7 (clone G043H7; BioLegend) for 10 min at 37°C. Additional surface stains were performed for 30 min at room temperature in the presence of Brilliant Stain Buffer Plus (BD Biosciences). Viable cells were identified by exclusion using a LIVE/DEAD Fixable Aqua Dead Cell Stain Kit (Thermo Fisher Scientific). Stained cells were washed in FACS buffer, fixed in PBS containing 1% PFA (Biotium), and acquired using a FACSymphony A5 (BD Biosciences). Flow cytometry reagents are listed in table S4.

### HCoV serology

Stored plasma samples were thawed on ice and centrifuged at 10,000*g* for 10 min. HCoV antibody concentrations were analyzed using a ProcartaPlex Human Coronavirus Ig Total 11-Plex Panel (Thermo Fisher Scientific). Fluorescence was determined using a Bio-Plex MAGPIX Multiplex Reader (Bio-Rad).

### Tetramer staining

Cryopreserved tonsil cells were thawed quickly, washed in PBS, distributed at 1 × 10^6^ to 2 × 10^6^ cells per well in 96-well U-bottom plates (Corning), and stained with anti–CCR7–APC-Cy7 (clone G043H7; BioLegend) for 10 min at 37°C. Cells were then labeled with a mix of BV421-conjugated human leukocyte antigen (HLA) class I tetramers for 15 min at room temperature in the presence of dasatinib (50 nM; STEMCELL Technologies). The following tetramers were used in these experiments, each corresponding to a defined epitope derived from EBV: GLCTLVAML/HLA-A*02:01, TYGPVFMCL/HLA-A*24:02, RPPIFIRRL/HLA-B*07:02, and RAKFKQLL/HLA-B*08:01 (*56*). Additional surface stains were performed for 30 min at 4°C. Viable cells were identified by exclusion using a LIVE/DEAD Fixable Aqua Dead Cell Stain Kit (Thermo Fisher Scientific). Stained cells were washed in FACS buffer, fixed in PBS containing with 1% PFA (Biotium), and acquired using a FACSymphony A5 (BD Biosciences). Flow cytometry reagents are listed in table S4.

### Data analysis and statistics

Flow cytometry data were analyzed using FlowJo software version 10.7.1 (FlowJo LLC). The gating strategy is shown in fig. S1. Net frequencies of virus-specific T cells were calculated by subtracting the frequency of specific marker^+^ T cells in the negative control from the frequency of specific marker^+^ T cells detected after stimulation with each peptide pool, with negative values set to 0. Stimulation indices were calculated as fold change. Positive responses required a stimulation index ≥2 and a minimum of five cells in the specific marker^+^ gate as described previously (*30*, *57*). Only responses assigned as positive based on these criteria were included in downstream analyses to limit the impact of background noise. Stimulation indices were only included if the calculations were based on >5 cells in each marker^+^ population. Dimensionality reduction was performed using the FlowJo plugin UMAP version 3.1 (FlowJo LLC). Down-sampled files concatenated from representative donors (*n* = 4) were used for these analyses with default settings (distance function: Euclidean; nearest neighbors: 15; minimum distance: 0.5) for the indicated markers (Fig. 3). Clusters of phenotypically related cells were identified using the FlowJo plugins Phenograph version 3.0 and ViolinBox version 5.1.8 (FlowJo LLC).

HCoV antibody concentrations were calculated using standard curves for relative quantitative results in units per milliliter using Bio-Plex Manager Software version 6.1 (Bio-Rad). Values below the standard range were set to the lowest standard concentration. The seropositivity threshold applied to all immunoglobulin (Ig) measurements was established using the following formula: mean concentration of SARS-CoV-2 trimer Ig for unexposed individuals + (3 * SD) (*58*), which provided an estimated specificity of 99%.

Statistical analyses were performed using Prism version 9 for Mac OS (GraphPad Software Inc.). Significance between two paired groups was assessed using the Wilcoxon signed-rank test, and significance between two unpaired groups was assessed using the Mann-Whitney test. Significance among three or more unpaired groups or among paired groups with missing values was assessed using the Kruskal-Wallis test with Dunn’s post test. Categorical variables were compared using Fisher’s exact test. Software programs are listed in table S4.

## Supplementary Material

20210914-1

## Notes

View/request a protocol for this paper from *Bio-protocol*.
