# Supplementary material for: Identification of resident memory CD8+ T cells with functional specificity for SARS-CoV-2 in unexposed oropharyngeal lymphoid tissue
Source: Sci Immunol. 2021 Oct 22;6(64):eabk0894. doi: 10.1126/sciimmunol.abk0894 (PMC10763663; doi:10.1126/sciimmunol.abk0894)
Supplement: 20210914-1 [file sciimmunol.abk0894.v1.pdf]

Cite as: J. Niessl *et al.*, *Sci. Immunol.* 10.1126/sciimmunol.abk0894 (2021).

CORONAVIRUS

Identification of resident memory CD8+ T cells with functional specificity for SARS-CoV-2 in unexposed oropharyngeal lymphoid tissue

Julia Niessl[1,*], Takuya Sekine[1], Joshua Lange[1], Viktoria Konya[1], Marianne Forkel[1,†], Jovana Maric[1], Anna Rao[1,‡], Luca Mazzurana[1], Efthymia Kokkinou[1], Whitney Weigel[1], Sian Llewellyn-Lacey[2], Emma B. Hodcroft[3,4], Annika C. Karlsson[5], Johan Fehrm[6,7], Joar Sundman[6,7], David A. Price[2,8], Jenny Mjösberg[1], Danielle Friberg[9], Marcus Buggert[1,*]

[1]Department of Medicine, Center for Infectious Medicine, Karolinska Institutet, Stockholm, Sweden. [2]Division of Infection and Immunity, Cardiff University School of Medicine, University Hospital of Wales, Cardiff, UK. [3]Biozentrum, University of Basel, Basel, Switzerland. [4]ISPM, University of Bern, Bern, Switzerland. [5]Division of Clinical Microbiology, Department of Laboratory Medicine, Karolinska Institutet, Stockholm, Sweden. [6]Department of Otorhinolaryngology, Karolinska University Hospital, Stockholm, Sweden. [7]Department of Clinical Science, Intervention and Technology (CLINTEC), Karolinska Institutet, Stockholm, Sweden. [8]Systems Immunity Research Institute, Cardiff University School of Medicine, University Hospital of Wales, Cardiff, UK. [9]Department of Surgical Sciences, Otorhinolaryngology and Head and Neck Surgery, Uppsala University, Uppsala, Sweden.

†Current affiliation: Roche Innovation Center, Basel, Switzerland.

‡Current affiliation: BioNTech SE, Mainz, Germany.

***Corresponding author. Email: julia.niessl@ki.se (J.N.) or marcus.buggert@ki.se (M.B.)**

Cross-reactive CD4+ T cells that recognize SARS-CoV-2 are more commonly detected in the peripheral blood of unexposed individuals compared to SARS-CoV-2-reactive CD8+ T cells. However, large numbers of memory CD8+ T cells reside in tissues, feasibly harboring localized SARS-CoV-2-specific immune responses. To test this idea, we performed a comprehensive functional and phenotypic analysis of virus-specific T cells in tonsils, a major lymphoid tissue site in the upper respiratory tract, and matched peripheral blood samples obtained from children and adults before the emergence of COVID-19. We found that SARS-CoV-2-specific memory CD4+ T cells could be found at similar frequencies in the tonsils and peripheral blood in unexposed individuals, whereas functional SARS-CoV-2-specific memory CD8+ T cells were almost only detectable in the tonsils. Tonsillar SARS-CoV-2-specific memory CD8+ T cells displayed a follicular homing and tissue-resident memory phenotype, similar to tonsillar Epstein-Barr virus-specific memory CD8+ T cells, but were functionally less potent than other virus-specific memory CD8+ T cell responses. The presence of pre-existing tissue-resident memory CD8+ T cells in unexposed individuals could potentially enable rapid sentinel immune responses against SARS-CoV-2.

INTRODUCTION

COVID-19 has caused millions of deaths worldwide, and hundreds of millions of people have been infected with the causative agent, SARS-CoV-2 (*1*). Large cohort studies have shown that increased age, being a male, and various comorbidities, such as obesity, cardiovascular disease, hypertension, and chronic lung diseases, associate with poor outcomes (*2*–*4*). Pre-existing immunity against SARS-CoV-2 may also modulate the severity of COVID-19 (*5*).

Several studies have reported the existence of functional T cell responses in the peripheral blood of SARS-CoV-2-unexposed individuals that cross-recognize SARS-CoV-2 (*6*–*14*). Cross-reactive SARS-CoV-2-specific memory (m)CD4+ T cells are readily detectable ex vivo in approximately 20–50% of unexposed people and become almost universally detectable after in vitro expansion (*6*), whereas cross-reactive SARS-CoV-2-specific mCD8+ T cells are much less common. These pre-existing cellular immune responses are likely generated by previous infections with common cold human coronaviruses (HCoVs), which share considerable sequence homology with SARS-CoV-2 (*12*), although other pathogens have also been implicated in this phenomenon (*15*, *16*).

Pre-existing T cell immunity associates with negative (*6*) and positive effects on the development of adaptive responses against SARS-CoV-2 (*17*, *18*). However, these studies are limited to analyses of peripheral blood samples, and cross-reactive T cells would likely need to be positioned at sites of viral entry to limit the severity of COVID-19. Immune surveillance is maintained by specialized T cells that recirculate between tissues and the intravascular space via the lymphatic system (*19*), whereas anatomically localized immunity is primarily a function of tissue-resident memory T (T_{RM}) cells (*20*), which

remain in situ and act as frontline defenders against pathogen invasion (*21*). T_{RM} cells constitutively express CD69 alone or in combination with CD103 and/or CD49a, all of which ensure tissue retention via different mechanisms (*22–24*). However, little is known about the function, phenotype, or even the existence of SARS-CoV-2-specific T_{RM} cells in unexposed individuals. This is an important knowledge gap in the light of growing evidence suggesting that T_{RM} cells are critical mediators of protection against viral diseases (*25–27*).

In this study, we conducted a flow cytometric survey of virus-specific T cell responses in oropharyngeal (tonsillar) lymphoid tissue and matched peripheral blood samples collected from children and adults before the onset of the current pandemic in 2019. Our results provide new information regarding the function, phenotype, and tissue compartmentalization of pre-existing mCD4$^+$ and mCD8$^+$ T cells directed against SARS-CoV-2, with potentially important implications for heterologous immune responses.

RESULTS

SARS-CoV-2-specific mCD8$^+$ T cells preferentially localize to oropharyngeal lymphoid tissue in unexposed individuals

To optimize assays for the detection of virus-specific mCD4$^+$ and mCD8$^+$ T cell responses in the upper respiratory tract, we first compared the expression of activation-induced markers (AIMs) on the cell surface (PD-L1, CD25, CD134/OX40, and CD137/4-1BB) or the intracellular expression of AIMs (4-1BB and CD154/CD40L) and cytokines (TNF-α and IFN-γ) in the absence or presence of virus peptide stimulation (Figs. S1 and S2). AIMs are dynamically regulated among circulating T cells and minimally expressed at baseline (*10*, *28–30*). In contrast, we found that AIMs were commonly expressed among tonsillar T cells in the absence of peptide stimulation (Fig. S2A–C), precluding the use of these markers alone for the reliable identification of infrequent antigen-specific responses (Fig. S2D, E), whereas the corresponding background levels of intracellular cytokines were negligible (Fig. S2A–C). We therefore used a combination of intracellular CD40L and TNF-α to identify mCD4$^+$ T cell responses and a combination of intracellular 4-1BB and IFN-γ to identify mCD8$^+$ T cell responses (Fig. S2D, E).

Tonsil samples were obtained from children (n=40) and adults (n=41) who underwent surgical intervention for obstructive sleep apnea at least one year before the emergence of COVID-19 (Table S1). Cells were stimulated with overlapping peptide pools spanning the spike, nucleocapsid, membrane, envelope, ORF1a, ORF1b, and ORF3–10 proteins of SARS-CoV-2 and selected immunodominant proteins encoded by Epstein-Barr virus (EBV), cytomegalovirus (CMV), or HCoV-OC43 (Fig. 1A, Fig. S3A). Antigen-specific mCD8$^+$ T cell responses directed against all four viruses were readily detected in tonsil samples, based on the concurrent up-regulation of intracellular 4-1BB and IFN-γ (Fig. 1A–D). SARS-CoV-2-specific mCD8$^+$ T cells occurred at lower frequencies compared with EBV-specific and CMV-specific mCD8$^+$ T cells (Fig. 1B, C) but were nonetheless detected in 26 of the 81 donors (32%) (Fig. 1D). In line with a lack of clear immunodominance patterns in peripheral blood samples (*10*, *11*), these tonsillar mCD8$^+$ T cell responses were directed against multiple proteins derived from SARS-CoV-2 (Fig. 1B–D). Tonsil samples commonly harbored mCD8$^+$ T cells specific for EBV (76%) and CMV (53%) but less commonly harbored mCD8$^+$ T cells specific for the seasonal HCoV-OC43 (28%) (Fig. 1D). Among tonsil samples with detectable mCD8$^+$ T cell responses against SARS-CoV-2, 50% showed reactivity against a single antigen, and <25% showed reactivity against more than two antigens (Fig. 1A, Fig. S3B). Stimulation indices were correlated positively among different SARS-CoV-2 antigens, and ORF1b-specific and/or ORF3–10-specific responses correlated positively with those directed against EBV, CMV, and HCoV-OC43 (Fig. 1E). The latter also demonstrated a positive correlation with responses directed against EBV and CMV (Fig. 1E).

Tonsillar mCD4$^+$ T cell responses directed against SARS-CoV-2 were detected in a subset of individuals, based on the concurrent up-regulation of intracellular CD40L and TNF-α (Fig. S4A–D). Higher background expression of these markers was observed among tonsillar mCD4$^+$ T cells compared with tonsillar mCD8$^+$ T cells in some individuals, likely reflecting greater levels of basal activation and effectively raising the detection threshold for antigen-specific responses (Figs. 1A and S4A). Despite these limitations, we detected SARS-CoV-2-reactive mCD4$^+$ T cell responses in 32% of tonsil samples (Fig. S4E), and in total, we detected SARS-CoV-2-reactive mCD4$^+$ and/or mCD8$^+$ T cell responses in 49% of tonsil samples (Fig. S4F).

Cross-reactive SARS-CoV-2-specific mCD4$^+$ T cell responses appear to be more common in peripheral blood than cross-reactive SARS-CoV-2-specific mCD8$^+$ T cell responses (*6–11*). Our analysis suggested SARS-CoV-2-specific mCD4$^+$ and mCD8$^+$ T cells were present at similar frequencies in SARS-CoV-2-unexposed oropharyngeal lymphoid tissue (Fig. S4F). To address this potential discrepancy, we compared virus-specific mCD4$^+$ and mCD8$^+$ T cell responses in matched tonsil and peripheral blood samples from a subset of donors in our cohort. SARS-CoV-2-specific mCD4$^+$ T cells were detected at similar frequencies in both compartments (Fig. S4G), whereas SARS-CoV-2-specific mCD8$^+$ T cells were detected almost exclusively in the tonsils (Figs. 1F–H). EBV-specific mCD8$^+$ T cells were also found predominantly in the tonsils (Fig. 1G), whereas CMV-specific T cells were mainly found in the circulation (Fig. 1G), as described previously (*31–33*). This approach enabled the sensitive detection of virus-specific CD4$^+$ and CD8$^+$ T cell responses, allowing us to show

that SARS-CoV-2-reactive mCD8+ T cells were preferentially localized to the tonsils in unexposed individuals.

SARS-CoV-2-specific mCD8+ T cells occur at similar frequencies and prevalence rates in oropharyngeal lymphoid tissue from unexposed children and adults

Pre-existing immunity may explain why children are less susceptible to severe COVID-19 (*34*). We therefore evaluated virus-specific tonsillar mCD8+ T cell responses in young children (age 2–5 years) and adults (age 28–67 years) (Fig. 2A). SARS-CoV-2-specific mCD8+ T cells were distributed similarly between groups in terms of frequency and overall prevalence, with the exception of responses directed against the spike protein, which occurred at lower magnitudes in children (Fig. 2B, C). EBV-specific mCD8+ T cells were less common in children (Fig. 2B), likely reflecting lower seroprevalence rates (*35*). Similarly, HCoV-OC43-specific mCD8+ T cells were undetectable in tonsil samples from children (Fig. 2B), consistent with lower seroprevalence rates for HCoVs-OC43 and other HCoVs (Fig. 2D, Fig. S5A, Table S2). Of note, HCoV-OC43-specific responses were also undetectable in some HCoV-OC43-seropositive adults (Fig. S5B), confirming the previously reported discordance between humoral and cellular immunity against HCoVs (*36*).

The presence of EBV-specific mCD8+ T cell responses associated with the occurrence of SARS-CoV-2-specific mCD8+ T cell responses in children but not in adults (Table S3). In addition, the stimulation indices for mCD8+ T cell responses directed against the SARS-CoV-2 nucleocapsid, ORF1b, and ORF3–10 proteins correlated positively with certain HCoV-specific antibody titers (Fig. 2E, Fig. S5C), and SARS-CoV-2-specific mCD8+ T cell responses were identified only in children with detectable antibodies specific for HKU-1 or NL63 and not in children with detectable antibodies specific for 229E or OC43 (Fig. S5D). No significant correlations were detected between age and SARS-CoV-2-derived antigen-specific mCD8+ T cell response stimulation indices in children or adults (Fig. 2D). However, positive correlations were detected between mCD8+ T cell responses directed against nucleocapsid, ORF1a, and ORF3–10 in children, and nucleocapsid only in adults, and the overall size of the mCD8+ T cell pool (Figs. 2E and S5C). Accordingly, previous exposure to other viruses, including HCoVs, not age, appeared to drive the formation of oropharyngeal mCD8+ T cell responses against SARS-CoV-2.

SARS-CoV-2-specific mCD8+ T cells exhibit a follicular homing and T_{RM} phenotype in oropharyngeal lymphoid tissue from unexposed individuals

To extend these findings, we investigated the phenotypic characteristics of virus-specific mCD8+ T cells in the tonsils, with a particular focus on markers of follicular homing and tissue residency. Importantly, the expression of surface markers in this analysis, including CD69, was not influenced by stimulation in these experiments (Fig. S6A–C). mCD8+ T cells specific for different SARS-CoV-2-derived antigens were pooled for downstream analyses on the grounds of phenotypic homogeneity (Fig. S6D, E).

Total and virus-specific mCD8+ T cells were concatenated from four representative individuals, and non-linear relationships among individual cells were assessed using Uniform Manifold Approximation and Projection (UMAP) (Fig. 3A). Distinct topographical regions were delineated by overlaying the expression of analyzed phenotypic markers, namely CD103, CD49a, CD69, CCR6, CXCR5, PD-1, CXCR3, CCR7, and CD45RA (Fig. 3B). Phenograph analysis revealed 11 unique clusters in the UMAP space (Fig. 3C), each with a distinct pattern of surface marker expression (Fig. 3D). Overlays of antigen-specific mCD8+ T cells revealed unique clustering based on specificity (Fig. 3E). SARS-CoV-2-specific and EBV-specific mCD8+ T cells were found mainly in clusters 1, 3, and 9 (Fig. 3G), which displayed high expression of the T_{RM} markers CD103 and CD69 and the follicular homing marker CXCR5 (Fig. 3B, D). In contrast, CMV-specific mCD8+ T cells were distributed among clusters 4 to 8, which included populations expressing high levels of CD45RA and populations lacking CD103 and CXCR5 (Fig. 3B–G). HCoV-OC43-specific mCD8+ T cells were found predominantly in cluster 2, which displayed high expression levels of the T_{RM} markers CD103, CD49a, and CD69. Manual gating analysis further confirmed these phenotypic differences among specificities with respect to CD103, CD69, and CXCR5 (Figs. 3H, I and S6F, G). SARS-CoV-2-specific and EBV-specific tonsillar mCD8+ T cells were therefore characterized by a follicular homing and T_{RM} phenotype, which was similar in children and adults (Fig. S6H, I).

Functional properties of virus-specific mCD8+ T cells in oropharyngeal lymphoid tissue

To assess the functionality of virus-specific tonsillar mCD8+ T cells, we quantified the surface mobilization of CD107a and the intracellular expression of TNF-α and IL-2 alongside 4-1BB and IFN-γ in response to peptide stimulation (Fig. 4A). Pre-existing mCD8+ T cells specific for SARS-CoV-2 mobilized CD107a and up-regulated TNF-α and IL-2 as individual functions less commonly than mCD8+ T cells specific for EBV, CMV, or HCoV-OC43 (Fig. 4B). In addition, mCD8+ T cells specific for EBV, CMV, or HCoV-OC43 were highly polyfunctional and frequently expressed IFN-γ together with CD107a, TNF-α, and/or IL-2 (Figs. 4C and S7A), whereas mCD8+ T cells specific for SARS-CoV-2 were generally oligofunctional (Figs. 4C and S7A) and expressed comparatively lower amounts of IFN-γ, TNF-α, and IL-2 on a per cell basis (Fig. 4D). A similar pattern of suboptimal functionality has been described previously for cross-reactive intravascular SARS-CoV-2-specific mCD4+ T cells, likely reflecting low-avidity recognition of the corresponding non-primary antigens (*6*).

Of note, mCD8⁺ T cells specific for each virus mobilized CD107a to a similar extent and produced largely equivalent amounts IFN-γ, TNF-α, and IL-2 on a per cell basis in children and adults (Fig. S7B). The functional profiles of EBV-specific and CMV-specific mCD8⁺ T cells were also similar in each group, although EBV-specific mCD8⁺ T cells more commonly produced IL-2 in adults (Fig. 4E). In contrast, SARS-CoV-2-specific mCD8⁺ T cells more commonly mobilized CD107a and more commonly expressed other functional markers alongside IFN-γ in children (Figs. 4E and S7C). Together, these data suggest that SARS-CoV-2-unexposed children harbored higher frequencies of polyfunctional SARS-CoV-2-reactive mCD8⁺ T cells in tonsillar lymphoid tissue compared with unexposed adults.

DISCUSSION

CD8⁺ T cells are essential for immune control and clearance of previously encountered viruses. CD8⁺ T cells may also provide a degree of heterologous protection against previously unencountered viruses expressing structurally related or unrelated antigens that elicit functional responses within the pre-existing memory pool as a consequence of cross-reactivity (*37–39*). In line with the concept of heterologous immunity, previous studies have demonstrated a high prevalence of SARS-CoV-2-specific mCD4⁺ T cells and a relative paucity of SARS-CoV-2-specific mCD8⁺ T cells in the peripheral blood of SARS-CoV-2-unexposed individuals (*8–14*). However, it has remained unclear to what extent such pre-existing T cell responses against SARS-CoV-2 exist in tissues, which is an important omission especially given the critical role of anatomically localized immunity in other viral infections (*25–27*). The present study was designed to address this knowledge gap and provide a comprehensive picture of pre-existing SARS-CoV-2-specific mCD4⁺ and mCD8⁺ T cell immunity in the tonsils, representing key lymphoid organs in the upper respiratory tract and the intravascular circulation. We found that SARS-CoV-2-specific mCD4⁺ T cells were distributed evenly between these compartments, whereas SARS-CoV-2-specific mCD8⁺ T cells were almost exclusively present in the tonsils. Earlier studies confined to analyses of peripheral blood samples have therefore likely underestimated the overall prevalence of heterologous mCD8⁺ T cell responses against SARS-CoV-2.

Tonsils play an important role in immune defense against inhaled or orally acquired pathogens, highlighted by the increased risk of upper respiratory tract infections in children after tonsillectomy (*40*). Although much emphasis has been placed on the nasal/respiratory route of viral transmission (*41*), the oral cavity, including the tonsils, can also be infected and potentially acts as a primary site for the acquisition of SARS-CoV-2 (*42*). We found that SARS-CoV-2-specific mCD8⁺ T cells expressed markers of tissue residency, potentially forming a sentinel immune response at a key site of viral entry (*27*). Importantly, T_{RM} cells function partly as innate-like sensors (*43*) and may therefore trigger an early type I IFN response, which commonly associates with non-severe forms of COVID-19. Of note, an early CD8⁺ T cell proliferation signature coinciding with a type I IFN response has been detected in asymptomatic and mild cases of COVID-19 before the detection of SARS-CoV-2 (*44*). This phenomenon associates with accelerated viral clearance and a less severe cytokine storm at the site of infection, as demonstrated for cross-reactive T_{RM} cell responses directed against influenza virus, SARS-CoV-1, or Middle East respiratory syndrome-related coronavirus in mice (*37*, *45*). It remains unclear whether such early activation of SARS-CoV-2-specific CD8⁺ T cells reflects pre-existing memory recall or rapid de novo priming from the naive pool in milder forms of COVID-19. However, recent infection with HCoVs associates with less severe disease and lower mortality rates after subsequent infection with SARS-CoV-2 (*17*). In addition, pre-existing virus-specific mCD4⁺ and mCD8⁺ T cells numerically expand in the peripheral blood after infection with SARS-CoV-2 (*46*, *47*), and more robust cellular and humoral immune responses occur after infection in patients with pre-existing CD4⁺ T cell responses directed against SARS-CoV-2 (*18*). Further studies incorporating larger numbers of patients, matched tissue samples, and longitudinal analyses will nonetheless be required to understand the full impact of pre-existing SARS-CoV-2-specific mCD4⁺ and mCD8⁺ T cell responses on the outcome of COVID-19.

EBV-specific mCD8⁺ T cells preferentially localized to the tonsils, where they exhibited a follicular homing and T_{RM} phenotype akin to SARS-CoV-2-specific mCD8⁺ T cells, whereas CMV-specific mCD8⁺ T cells, which mostly lacked T_{RM} markers, are largely confined to the intravascular circulation (*33*). This anatomical dichotomy makes sense biologically, given the distinct tropism patterns of EBV and CMV with EBV being transmitted orally and mainly infecting B lymphocytes residing in lymphoid tissues and epithelial cells and CMV spreading throughout the body to infect multiple cell types and organs. CD69⁺CD103⁺ tonsillar mCD8⁺ T cells are located near the epithelial barrier surface, likely facilitated by high expression levels of E-cadherin (*33*). Accordingly, the pre-existing SARS-CoV-2-specific tonsillar mCD8⁺ T cells identified here might have been positioned near sites of viral entry, potentially enabling them to spearhead an early immune response.

Children and young adults are less prone to severe COVID-19 than older adults (*48*). Although there is a decline in cross-reactive SARS-CoV-2-specific humoral and cellular immunity with age (*49*, *50*), we found that pre-existing SARS-CoV-2-specific tonsillar mCD8⁺ T cells in children and adults were similar in terms of frequency, phenotype, and overall prevalence. It is notable that we did not detect HCoV-OC43-specific mCD8⁺ T cell responses in tonsils obtained from

children, despite the fact that some of these individuals were HCoV-OC43-seropositive and/or harbored detectable $mCD4^+$ T cell responses directed against HCoV-OC43. This discrepancy could reflect sensitivity limitations in our approach to the detection of antigen-specific $mCD8^+$ T cells directly ex vivo. In addition, HCoV-specific $mCD8^+$ T cell responses may be liable to decay (*51*), and repeated viral exposure may be necessary to induce long-lived HCoV-specific $CD8^+$ T_{RM} cells (*52*). A similar discrepancy is reported in a study of peripheral blood samples comparing cellular and humoral responses against HCoVs (*36*).

It is likely that pre-existing SARS-CoV-2-specific $mCD4^+$ and $mCD8^+$ T cell immunity is generated via previous encounters with HCoVs. In line with this notion, we found that SARS-CoV-2-specific $mCD8^+$ T cell response frequencies correlated with certain HCoV-specific antibody titers, and in children, we identified SARS-CoV-2-specific $mCD8^+$ T cell responses almost exclusively in individuals with detectable antibodies specific for HCoV-HKU-1 or HCoV-NL63. However, we also found that the presence of EBV-specific $mCD8^+$ T cell responses in children, likely reflecting persistent infection, associated with the occurrence of SARS-CoV-2-specific $mCD8^+$ T cell responses potentially arising as a consequence of heterologous immunity (*38*). Alternatively, the presence of EBV-specific $mCD8^+$ T cell responses could identify children with greater overall levels of exposure to endemic pathogens. Consistent with either possibility, the stimulation indices for some SARS-CoV-2-specific $mCD8^+$ T cell responses correlated with the overall size of the $mCD8^+$ T cell pool, which provides a general measure of immunological experience (*6*). Accordingly, prior antigen experience is likely the key driver of heterologous immunity, irrespective of the precise mechanism, shaping a diverse repertoire of $mCD8^+$ T cells incorporating various specificities with the ability to cross-recognize SARS-CoV-2.

Overall, we detected SARS-CoV-2-specific $mCD4^+$ T cell responses in 29% of unexposed peripheral blood samples, which is within the range reported previously (*8*–*14*). However, the true prevalence may be higher, because recovered cell numbers in some cases precluded a full assessment of all viral antigen specificities. A similar caveat applies to the identification of SARS-CoV-2-specific $mCD8^+$ T cell responses in peripheral blood samples and the identification of SARS-CoV-2-specific $mCD4^+$ and $mCD8^+$ T cell responses in the tonsils. In addition, our study was limited by detection based on the production of IFN-γ or TNF-α, which excluded cells that did not produce these cytokines in response to antigen encounter. Furthermore, a high level of background activation without any peptide stimulation in some individuals may prevent the detection of low frequency virus-specific T cell responses. It should also be noted that our findings cannot be taken in isolation as evidence of heterologous immune protection against SARS-CoV-2.

In summary, we have shown that SARS-CoV-2-unexposed children and adults commonly harbor tonsillar $CD8^+$ T_{RM} cells that react with SARS-CoV-2. Additional studies are now warranted to determine if these pre-existing $CD8^+$ T_{RM} cells lead to early viral containment, potentially mitigating the course of COVID-19.

MATERIALS AND METHODS

Study design

The objective of this study was to characterize SARS-CoV-2-reactive $mCD4^+$ and $mCD8^+$ T cell responses in tonsillar lymphoid tissue and matched peripheral blood samples obtained from children and adults prior to the current pandemic between 2015 and 2018. Functional and phenotypic analyses were extended for comparative purposes to include $mCD4^+$ and $mCD8^+$ T cell responses elicited by natural infections with EBV, CMV, and HCoV-OC43.

Human subjects and ethics

Tonsil samples and matched peripheral blood mononuclear cells (PBMCs) were obtained from individuals undergoing tonsillectomy for obstructive sleep apnea at the Department of Otorhinolaryngology, Karolinska University Hospital, Stockholm, Sweden. All samples were collected between 2015 and 2018. The study was approved by the regional ethics committee in Stockholm (2014/1000-31 for the child cohort and 2015/755-31, 2016/128-32, and 2017/2275-32 for the adult cohort). Written informed consent was obtained from all participants or their legal representatives in accordance with the Declaration of Helsinki. Whole tonsils were cut into small pieces, ground through a cell strainer (pore size = 100 μm), filtered through another cell strainer (pore size = 40 μm), and resuspended in phosphate-buffered saline (PBS). Mononuclear cells were isolated via standard density gradient centrifugation and cryopreserved in fetal bovine serum (FBS) containing 10% dimethyl sulfoxide (DMSO). PBMCs were isolated and cryopreserved similarly. Plasma samples were collected from some individuals (n = 21 children and n = 12 adults) and stored at −80°C. The adult tonsil cohort was dominated by male participants in this study, because obstructive sleep apnea is more common in adult men than in adult women (*53*). Plasma samples were also collected from two convalescent individuals recovering from infection with SARS-CoV-2 (Ethical approval: dnr 2019-05757 and dnr 2014/1000-31/1) and stored at −80°C. Donor characteristics are summarized in Table S1.

Peptides

Lyophilized pools of overlapping peptides spanning the relevant proteins were reconstituted in DMSO and further diluted to 50 or 100 μg/ml in PBS. Reconstituted pools were aliquoted and stored at −20°C. Overlapping peptide pools spanning the SARS-CoV-2 spike (Prot_S) and nucleocapsid (Prot_N) proteins were obtained from Miltenyi Biotec.

Overlapping peptide pools spanning the SARS-CoV-2 membrane and envelope (Membrane+Env) proteins and the ORF3A, ORF6, ORF7A, ORF8, and ORF10 (ORF3–10) proteins were synthesized using sequences derived from GenBank reference MT093571.1 (Sigma-Aldrich). Overlapping peptide pools spanning the SARS-CoV-2 ORF1a and ORF1b proteins were generated by combining NSP1–11 and NSP12–16, respectively (JPT Peptide Technologies). Overlapping peptide pools spanning the EBV BZLF1, EBNA-1, EBNA-3a, and LMP2 proteins were purchased from JPT Peptide Technologies. Overlapping peptide pools spanning the CMV pp65 and IE-1 proteins were purchased from Peptides & Elephants. Metadata for all available OC43 sequences on ViPR were downloaded on January 27, 2020. Sequences were filtered to include isolates from humans and exclude isolates from cell passage or vaccine development studies. One sequence per country per year was selected to create a representative set of OC43 sequences. The spike, nucleocapsid, and membrane gene amino acid translations (as available) were downloaded from GenBank. The amino acid sequences for each gene were then translated using the '–auto' setting in MAFFT (*54*), and a consensus sequence for each gene was generated using the 'dump_consensus()' method with default settings in BioPython (*55*). Overlapping peptide pools spanning these HCoV-OC43 spike, nucleocapsid, and membrane (OC43) protein consensus sequences were synthesized by Sigma-Aldrich. The relevant peptides were combined to generate single pools for EBV, CMV, and HCoV-OC43.

Intracellular cytokine staining

Cryopreserved tonsil cells and PBMCs were thawed quickly, resuspended in RPMI 1640 containing 10% FBS, 1% L-glutamine, and 1% penicillin/streptomycin (complete medium) in the presence of DNase I (10 U/ml; Sigma-Aldrich), and rested at 1–2 × 10^6 cells/well in 96-well U-bottom plates (Corning) for 5 hours at 37°C. The medium was then supplemented with anti-CXCR5–BB515 (clone RF8B2; BD Biosciences), followed 15 min later by the relevant peptide pools (0.5 μg/ml/peptide), and a further 1 hour later by brefeldin A (1 μg/ml; Sigma-Aldrich), monensin (0.7 μg/ml; BD Biosciences), and anti-CD107a–BV785 (clone H4A3; BioLegend). In one experiment, tonsil cells were stained with anti-CD69–BUV563 (clone FN50; BD Biosciences) for 30 min at room temperature and washed in complete medium before stimulation (Fig. S6A). Negative control wells contained equivalent DMSO, and positive control wells contained staphylococcal enterotoxin B (SEB; 0.5 μg/ml; Sigma-Aldrich). After 9 hours, cells were washed in PBS supplemented with 2% FBS and 2 mM EDTA (FACS buffer) and stained with anti-CCR4–BB700 (clone 1G1; BD Biosciences), anti-CCR6–BUV737 (clone 11A9; BD Biosciences), anti-CCR7–APC-Cy7 (clone G043H7; BioLegend), and anti-CXCR3–BV750 (clone 1C6; BD Biosciences) for 10 min at 37°C. Additional surface stains were performed for 30 min at room temperature in the presence of Brilliant Stain Buffer Plus (BD Biosciences). Viable cells were identified by exclusion using a LIVE/DEAD Fixable Aqua Dead Cell Stain Kit (Thermo Fisher Scientific). Cells were then washed in FACS buffer and fixed/permeabilized using a FoxP3 Transcription Factor Staining Buffer Set (Thermo Fisher Scientific). Intracellular stains were performed for 30 min at room temperature. Stained cells were washed in FACS buffer, fixed in PBS containing 1% paraformaldehyde (PFA; Biotium), and acquired using a FACSymphony A5 (BD Biosciences). Flow cytometry reagents are listed in Table S4.

Surface staining for activation-induced markers

Cryopreserved tonsil cells and PBMCs were thawed quickly, resuspended in complete medium in the presence of DNase I (10 U/ml; Sigma-Aldrich), and rested at 1–2 × 10^6 cells/well in 96-well U-bottom plates (Corning) for 5 hours at 37°C. The medium was then supplemented with the relevant peptide pools (0.5 μg/ml/peptide). Negative control wells contained equivalent DMSO, and positive control wells contained staphylococcal enterotoxin B (SEB; 0.5 μg/ml; Sigma-Aldrich). After 18 hours, cells were washed in FACS buffer and stained with anti-CCR7–APC-Cy7 (clone G043H7; BioLegend) for 10 min at 37°C. Additional surface stains were performed for 30 min at room temperature in the presence of Brilliant Stain Buffer Plus (BD Biosciences). Viable cells were identified by exclusion using a LIVE/DEAD Fixable Aqua Dead Cell Stain Kit (Thermo Fisher Scientific). Stained cells were washed in FACS buffer, fixed in PBS containing 1% PFA (Biotium), and acquired using a FACSymphony A5 (BD Biosciences). Flow cytometry reagents are listed in Table S4.

Human coronavirus serology

Stored plasma samples were thawed on ice and centrifuged at 10,000 × g for 10 min. Human coronavirus antibody concentrations were analyzed using a ProcartaPlex Human Coronavirus Ig Total 11-Plex Panel (Thermo Fisher Scientific). Fluorescence was determined using a Bio-Plex MAGPIX Multiplex Reader (Bio-Rad).

Tetramer staining

Cryopreserved tonsil cells were thawed quickly, washed in PBS, distributed at 1–2 × 10^6 cells/well in 96-well U-bottom plates (Corning), and stained with anti-CCR7–APC-Cy7 (clone G043H7; BioLegend) for 10 min at 37°C. Cells were then labeled with a mix of BV421-conjugated human leukocyte antigen (HLA) class I tetramers for 15 min at room temperature in the presence of dasatinib (50 nM; STEMCELL). The following tetramers were used in these experiments, each corresponding to a defined epitope derived from EBV: GLCTLVAML/HLA-A*02:01, TYGPVFMCL/HLA-A*24:02, RPPIFIRRL/HLA-B*07:02, and RAKFKQLL/HLA-B*08:01 (*56*). Additional surface stains were performed for 30 min at 4°C. Viable cells were identified by exclusion using a LIVE/DEAD Fixable Aqua Dead Cell Stain Kit (Thermo Fisher

Scientific). Stained cells were washed in FACS buffer, fixed in PBS containing with 1% PFA (Biotium), and acquired using a FACSymphony A5 (BD Biosciences). Flow cytometry reagents are listed in Table S4.

Data analysis and statistics

Flow cytometry data were analyzed using FlowJo software version 10.7.1 (FlowJo LLC). The gating strategy is shown in Fig. S1. Net frequencies of virus-specific T cells were calculated by subtracting the frequency of specific marker$^+$ T cells in the negative control from the frequency of specific marker$^+$ T cells detected after stimulation with each peptide pool, with negative values set to 0. Stimulation indices were calculated as fold change. Positive responses required a stimulation index ≥2 and a minimum of 5 cells in the specific marker$^+$ gate as described previously (*30*, *57*). Only responses assigned as positive based on these criteria were included in downstream analyses to limit the impact of background noise. Stimulation indices were only included if the calculations were based on >5 cells in each marker$^+$ population. Dimensionality reduction was performed using the FlowJo plugin UMAP version 3.1 (FlowJo LLC). Downsampled files concatenated from representative donors (n=4) were used for these analyses with default settings (distance function: Euclidean; nearest neighbors: 15; minimum distance: 0.5) for the indicated markers (Fig. 3). Clusters of phenotypically related cells were identified using the FlowJo plugins PhenoGraph version 3.0 and Violinbox version 5.1.8 (FlowJo LLC).

Human coronavirus antibody concentrations were calculated using standard curves for relative quantitative results in units/ml using Bio-Plex Manager Software version 6.1 (Bio-Rad). Values below the standard range were set to the lowest standard concentration. The seropositivity threshold applied to all Ig measurements was established using the following formula: mean concentration of SARS-CoV-2 trimer Ig for unexposed individuals + (3 * standard deviation) (*58*), which provided an estimated specificity of 99%.

Statistical analyses were performed using Prism version 9 for macOS (GraphPad Software Inc.). Significance between two paired groups was assessed using the Wilcoxon signed rank test, and significance between two unpaired groups was assessed using the Mann-Whitney test. Significance among three or more unpaired groups or among paired groups with missing values was assessed using the Kruskal-Wallis test with Dunn's post-test. Categorical variables were compared using Fisher's exact test. Software programs are listed in Table S4.

SUPPLEMENTARY MATERIALS

www.science.org/doi/10.1126/sciimmunol.abk0894

Figs. S1 to S7

Tables S1 to S5

REFERENCES AND NOTES

1. WHO Coronavirus (COVID-19) Dashboard (available at https://covid19.who.int).
2. E. J. Williamson, A. J. Walker, K. Bhaskaran, S. Bacon, C. Bates, C. E. Morton, H. J. Curtis, A. Mehrkar, D. Evans, P. Inglesby, J. Cockburn, H. I. McDonald, B. MacKenna, L. Tomlinson, I. J. Douglas, C. T. Rentsch, R. Mathur, A. Y. S. Wong, R. Grieve, D. Harrison, H. Forbes, A. Schultze, R. Croker, J. Parry, F. Hester, S. Harper, R. Perera, S. J. W. Evans, L. Smeeth, B. Goldacre, Factors associated with COVID-19-related death using OpenSAFELY. *Nature* **584**, 430–436 (2020). doi:10.1038/s41586-020-2521-4 Medline
3. W. J. Guan, W. H. Liang, Y. Zhao, H. R. Liang, Z. S. Chen, Y. M. Li, X. Q. Liu, R. C. Chen, C. L. Tang, T. Wang, C. Q. Ou, L. Li, P. Y. Chen, L. Sang, W. Wang, J. F. Li, C. C. Li, L. M. Ou, B. Cheng, S. Xiong, Z. Y. Ni, J. Xiang, Y. Hu, L. Liu, H. Shan, C. L. Lei, Y. X. Peng, L. Wei, Y. Liu, Y. H. Hu, P. Peng, J. M. Wang, J. Y. Liu, Z. Chen, G. Li, Z. J. Zheng, S. Q. Qiu, J. Luo, C. J. Ye, S. Y. Zhu, L. L. Cheng, F. Ye, S. Y. Li, J. P. Zheng, N. F. Zhang, N. S. Zhong, J. X. He; China Medical Treatment Expert Group for COVID-19, Comorbidity and its impact on 1590 patients with COVID-19 in China: A nationwide analysis. *Eur. Respir. J.* **55**, 2000547 (2020). doi:10.1183/13993003.00547-2020 Medline
4. H. Peckham, N. M. de Gruijter, C. Raine, A. Radziszewska, C. Ciurtin, L. R. Wedderburn, E. C. Rosser, K. Webb, C. T. Deakin, Male sex identified by global COVID-19 meta-analysis as a risk factor for death and ITU admission. *Nat. Commun.* **11**, 6317 (2020). doi:10.1038/s41467-020-19741-6 Medline
5. M. Lipsitch, Y. H. Grad, A. Sette, S. Crotty, Cross-reactive memory T cells and herd immunity to SARS-CoV-2. *Nat. Rev. Immunol.* **20**, 709–713 (2020). doi:10.1038/s41577-020-00460-4 Medline
6. P. Bacher, E. Rosati, D. Esser, G. R. Martini, C. Saggau, E. Schiminsky, J. Dargvainiene, I. Schröder, I. Wieters, Y. Khodamoradi, F. Eberhardt, M. J. G. T. Vehreschild, H. Neb, M. Sonntagbauer, C. Conrad, F. Tran, P. Rosenstiel, R. Markewitz, K.-P. Wandinger, M. Augustin, J. Rybniker, M. Kochanek, F. Leypoldt, O. A. Cornely, P. Koehler, A. Franke, A. Scheffold, Low-Avidity CD4$^+$ T Cell Responses to SARS-CoV-2 in Unexposed Individuals and Humans with Severe COVID-19. *Immunity* **53**, 1258–1271.e5 (2020). doi:10.1016/j.immuni.2020.11.016 Medline
7. T. Sekine, A. Perez-Potti, O. Rivera-Ballesteros, K. Strålin, J.-B. Gorin, A. Olsson, S. Llewellyn-Lacey, H. Kamal, G. Bogdanovic, S. Muschiol, D. J. Wullimann, T. Kammann, J. Emgård, T. Parrot, E. Folkesson, O. Rooyackers, L. I. Eriksson, J.-I. Henter, A. Sönnerborg, T. Allander, J. Albert, M. Nielsen, J. Klingström, S. Gredmark-Russ, N. K. Björkström, J. K. Sandberg, D. A. Price, H.-G. Ljunggren, S. Aleman, M. Buggert; Karolinska COVID-19 Study Group, Robust T Cell Immunity in Convalescent Individuals with Asymptomatic or Mild COVID-19. *Cell* **183**, 158–168.e14 (2020). doi:10.1016/j.cell.2020.08.017 Medline
8. D. Weiskopf, K. S. Schmitz, M. P. Raadsen, A. Grifoni, N. M. A. Okba, H. Endeman, J. P. C. van den Akker, R. Molenkamp, M. P. G. Koopmans, E. C. M. van Gorp, B. L. Haagmans, R. L. de Swart, A. Sette, R. D. de Vries, Phenotype and kinetics of SARS-CoV-2-specific T cells in COVID-19 patients with acute respiratory distress syndrome. *Sci. Immunol.* **5**, eabd2071 (2020). doi:10.1126/sciimmunol.abd2071 Medline
9. A. Nelde, T. Bilich, J. S. Heitmann, Y. Maringer, H. R. Salih, M. Roerden, M. Lübke, J. Bauer, J. Rieth, M. Wacker, A. Peter, S. Hörber, B. Traenkle, P. D. Kaiser, U. Rothbauer, M. Becker, D. Junker, G. Krause, M. Strengert, N. Schneiderhan-Marra, M. F. Templin, T. O. Joos, D. J. Kowalewski, V. Stos-Zweifel, M. Fehr, A. Rabsteyn, V. Mirakaj, J. Karbach, E. Jäger, M. Graf, L.-C. Gruber, D. Rachfalski, B. Preuß, I. Hagelstein, M. Märklin, T. Bakchoul, C. Gouttefangeas, O. Kohlbacher, R. Klein, S. Stevanović, H.-G. Rammensee, J. S. Walz, SARS-CoV-2-derived peptides define heterologous and COVID-19-induced T cell recognition. *Nat. Immunol.* **22**, 74–85 (2021). doi:10.1038/s41590-020-00808-x Medline
10. A. Grifoni, D. Weiskopf, S. I. Ramirez, J. Mateus, J. M. Dan, C. R. Moderbacher, S. A. Rawlings, A. Sutherland, L. Premkumar, R. S. Jadi, D. Marrama, A. M. de Silva, A. Frazier, A. F. Carlin, J. A. Greenbaum, B. Peters, F. Krammer, D. M. Smith, S. Crotty, A. Sette, Targets of T Cell Responses to SARS-CoV-2 Coronavirus in Humans with COVID-19 Disease and Unexposed Individuals. *Cell* **181**, 1489–1501.e15 (2020). doi:10.1016/j.cell.2020.05.015 Medline
11. N. Le Bert, A. T. Tan, K. Kunasegaran, C. Y. L. Tham, M. Hafezi, A. Chia, M. H. Y. Chng, M. Lin, N. Tan, M. Linster, W. N. Chia, M. I.-C. Chen, L.-F. Wang, E. E. Ooi, S. Kalimuddin, P. A. Tambyah, J. G.-H. Low, Y.-J. Tan, A. Bertoletti, SARS-CoV-2-specific T cell immunity in cases of COVID-19 and SARS, and uninfected controls. *Nature* **584**, 457–462 (2020). doi:10.1038/s41586-020-2550-z Medline

12. J. Mateus, A. Grifoni, A. Tarke, J. Sidney, S. I. Ramirez, J. M. Dan, Z. C. Burger, S. A. Rawlings, D. M. Smith, E. Phillips, S. Mallal, M. Lammers, P. Rubiro, L. Quiambao, A. Sutherland, E. D. Yu, R. da Silva Antunes, J. Greenbaum, A. Frazier, A. J. Markmann, L. Premkumar, A. de Silva, B. Peters, S. Crotty, A. Sette, D. Weiskopf, Selective and cross-reactive SARS-CoV-2 T cell epitopes in unexposed humans. *Science* **370**, 89–94 (2020). doi:10.1126/science.abd3871 Medline
13. B. J. Meckiff, C. Ramírez-Suástegui, V. Fajardo, S. J. Chee, A. Kusnadi, H. Simon, S. Eschweiler, A. Grifoni, E. Pelosi, D. Weiskopf, A. Sette, F. Ay, G. Seumois, C. H. Ottensmeier, P. Vijayanand, Imbalance of Regulatory and Cytotoxic SARS-CoV-2-Reactive CD4+ T Cells in COVID-19. *Cell* **183**, 1340–1353.e16 (2020). doi:10.1016/j.cell.2020.10.001 Medline
14. J. Braun, L. Loyal, M. Frentsch, D. Wendisch, P. Georg, F. Kurth, S. Hippenstiel, M. Dingeldey, B. Kruse, F. Fauchere, E. Baysal, M. Mangold, L. Henze, R. Lauster, M. A. Mall, K. Beyer, J. Röhmel, S. Voigt, J. Schmitz, S. Miltenyi, I. Demuth, M. A. Müller, A. Hocke, M. Witzenrath, N. Suttorp, F. Kern, U. Reimer, H. Wenschuh, C. Drosten, V. M. Corman, C. Giesecke-Thiel, L. E. Sander, A. Thiel, SARS-CoV-2-reactive T cells in healthy donors and patients with COVID-19. *Nature* **587**, 270–274 (2020). doi:10.1038/s41586-020-2598-9 Medline
15. P. A. Reche, Potential Cross-Reactive Immunity to SARS-CoV-2 From Common Human Pathogens and Vaccines. *Front. Immunol.* **11**, 586984 (2020). doi:10.3389/fimmu.2020.586984 Medline
16. U. Stervbo, S. Rahmann, T. Roch, T. H. Westhoff, N. Babel, Epitope similarity cannot explain the pre-formed T cell immunity towards structural SARS-CoV-2 proteins. *Sci. Rep.* **10**, 18995 (2020). doi:10.1038/s41598-020-75972-z Medline
17. M. Sagar, K. Reifler, M. Rossi, N. S. Miller, P. Sinha, L. F. White, J. P. Mizgerd, Recent endemic coronavirus infection is associated with less-severe COVID-19. *J. Clin. Invest.* **131**, e143380 (2021). doi:10.1172/JCI143380 Medline
18. L. Loyal, J. Braun, L. Henze, B. Kruse, M. Dingeldey, U. Reimer, F. Kern, T. Schwarz, M. Mangold, C. Unger, F. Dörfler, S. Kadler, J. Rosowski, K. Gürcan, Z. Uyar-Aydin, M. Frentsch, F. Kurth, K. Schnatbaum, M. Eckey, S. Hippenstiel, A. Hocke, M. A. Müller, B. Sawitzki, S. Miltenyi, F. Paul, M. A. Mall, H. Wenschuh, S. Voigt, C. Drosten, R. Lauster, N. Lachman, L.-E. Sander, V. M. Corman, J. Röhmel, L. Meyer-Arndt, A. Thiel, C. Giesecke-Thiel, Cross-reactive CD4+ T cells enhance SARS-CoV-2 immune responses upon infection and vaccination. *Science* eabh1823 (2021). doi:10.1126/science.abh1823 Medline
19. M. Buggert, L. A. Vella, S. Nguyen, V. H. Wu, Z. Chen, T. Sekine, A. Perez-Potti, C. R. Maldini, S. Manne, S. Darko, A. Ransier, L. Kuri-Cervantes, A. S. Japp, I. B. Brody, M. A. Ivarsson, J.-B. Gorin, O. Rivera-Ballesteros, L. Hertwig, J. P. Antel, M. E. Johnson, A. Okoye, L. Picker, G. Vahedi, E. Sparrelid, S. Llewellyn-Lacey, E. Gostick, J. K. Sandberg, N. Björkström, A. Bar-Or, Y. Dori, A. Naji, D. H. Canaday, T. M. Laufer, A. D. Wells, D. A. Price, I. Frank, D. C. Douek, E. J. Wherry, M. G. Itkin, M. R. Betts, The Identity of Human Tissue-Emigrant CD8+ T Cells. *Cell* **183**, 1946–1961.e15 (2020). doi:10.1016/j.cell.2020.11.019 Medline
20. E. M. Steinert, J. M. Schenkel, K. A. Fraser, L. K. Beura, L. S. Manlove, B. Z. Igyártó, P. J. Southern, D. Masopust, Quantifying Memory CD8 T Cells Reveals Regionalization of Immunosurveillance. *Cell* **161**, 737–749 (2015). doi:10.1016/j.cell.2015.03.031 Medline
21. J. M. Schenkel, D. Masopust, Tissue-resident memory T cells. *Immunity* **41**, 886–897 (2014). doi:10.1016/j.immuni.2014.12.007 Medline
22. E. C. Reilly, K. Lambert Emo, P. M. Buckley, N. S. Reilly, I. Smith, F. A. Chaves, H. Yang, P. W. Oakes, D. J. Topham, T_{RM} integrins CD103 and CD49a differentially support adherence and motility after resolution of influenza virus infection. *Proc. Natl. Acad. Sci. U.S.A.* **117**, 12306–12314 (2020). doi:10.1073/pnas.1915681117 Medline
23. S. K. Bromley, H. Akbaba, V. Mani, R. Mora-Buch, A. Y. Chasse, A. Sama, A. D. Luster, CD49a Regulates Cutaneous Resident Memory CD8+ T Cell Persistence and Response. *Cell Rep.* **32**, 108085 (2020). doi:10.1016/j.celrep.2020.108085 Medline
24. L. K. Mackay, A. Braun, B. L. Macleod, N. Collins, C. Tebartz, S. Bedoui, F. R. Carbone, T. Gebhardt, Cutting edge: CD69 interference with sphingosine-1-phosphate receptor function regulates peripheral T cell retention. *J. Immunol.* **194**, 2059–2063 (2015). doi:10.4049/jimmunol.1402256 Medline
25. T. Gebhardt, L. M. Wakim, L. Eidsmo, P. C. Reading, W. R. Heath, F. R. Carbone, Memory T cells in nonlymphoid tissue that provide enhanced local immunity during infection with herpes simplex virus. *Nat. Immunol.* **10**, 524–530 (2009). doi:10.1038/ni.1718 Medline
26. X. Jiang, R. A. Clark, L. Liu, A. J. Wagers, R. C. Fuhlbrigge, T. S. Kupper, Skin infection generates non-migratory memory CD8+ T(RM) cells providing global skin immunity. *Nature* **483**, 227–231 (2012). doi:10.1038/nature10851 Medline
27. A. Pizzolla, T. H. O. Nguyen, J. M. Smith, A. G. Brooks, K. Kedzieska, W. R. Heath, P. C. Reading, L. M. Wakim, Resident memory CD8+ T cells in the upper respiratory tract prevent pulmonary influenza virus infection. *Sci. Immunol.* **2**, eaam6970 (2017). doi:10.1126/sciimmunol.aam6970 Medline
28. J. Niessl, A. E. Baxter, P. Mendoza, M. Jankovic, Y. Z. Cohen, A. L. Butler, C.-L. Lu, M. Dubé, I. Shimeliovich, H. Gruell, F. Klein, M. Caskey, M. C. Nussenzweig, D. E. Kaufmann, Combination anti-HIV-1 antibody therapy is associated with increased virus-specific T cell immunity. *Nat. Med.* **26**, 222–227 (2020). doi:10.1038/s41591-019-0747-1 Medline
29. J. M. Dan, C. S. Lindestam Arlehamn, D. Weiskopf, R. da Silva Antunes, C. Havenar-Daughton, S. M. Reiss, M. Brigger, M. Bothwell, A. Sette, S. Crotty, A cytokine-independent approach to identify antigen-specific human germinal center Tfh cells and rare antigen-specific CD4+ T cells in blood. *J. Immunol.* **197**, 983–993 (2016). doi:10.4049/jimmunol.1600318 Medline
30. S. Reiss, A. E. Baxter, K. M. Cirelli, J. M. Dan, A. Morou, A. Daigneault, N. Brassard, G. Silvestri, J.-P. Routy, C. Havenar-Daughton, S. Crotty, D. E. Kaufmann, Comparative analysis of activation induced marker (AIM) assays for sensitive identification of antigen-specific CD4 T cells. *PLOS ONE* **12**, e0186998 (2017). doi:10.1371/journal.pone.0186998 Medline
31. M. Buggert, S. Nguyen, G. Salgado-Montes de Oca, B. Bengsch, S. Darko, A. Ransier, E. R. Roberts, D. Del Alcazar, I. B. Brody, L. A. Vella, L. Beura, S. Wijeyesinghe, R. S. Herati, P. M. Del Rio Estrada, Y. Ablanedo-Terrazas, L. Kuri-Cervantes, A. Sada Japp, S. Manne, S. Vartanian, A. Huffman, J. K. Sandberg, E. Gostick, G. Nadolski, G. Silvestri, D. H. Canaday, D. A. Price, C. Petrovas, L. F. Su, G. Vahedi, Y. Dori, I. Frank, M. G. Itkin, E. J. Wherry, S. G. Deeks, A. Naji, G. Reyes-Terán, D. Masopust, D. C. Douek, M. R. Betts, Identification and characterization of HIV-specific resident memory CD8+ T cells in human lymphoid tissue. *Sci. Immunol.* **3**, eaar4526 (2018). doi:10.1126/sciimmunol.aar4526 Medline
32. A. D. Hislop, M. Kuo, A. B. Drake-Lee, A. N. Akbar, W. Bergler, N. Hammerschmitt, N. Khan, U. Palendira, A. M. Leese, J. M. Timms, A. I. Bell, C. D. Buckley, A. B. Rickinson, Tonsillar homing of Epstein-Barr virus-specific CD8+ T cells and the virus-host balance. *J. Clin. Invest.* **115**, 2546–2555 (2005). doi:10.1172/JCI24810 Medline
33. H. G. Woon, A. Braun, J. Li, C. Smith, J. Edwards, F. Sierro, C. G. Feng, R. Khanna, M. Elliot, A. Bell, A. D. Hislop, S. G. Tangye, A. B. Rickinson, T. Gebhardt, W. J. Britton, U. Palendira, Compartmentalization of Total and Virus-Specific Tissue-Resident Memory CD8+ T Cells in Human Lymphoid Organs. *PLOS Pathog.* **12**, e1005799 (2016). doi:10.1371/journal.ppat.1005799 Medline
34. J. B. Steinman, F. M. Lum, P. P.-K. Ho, N. Kaminski, L. Steinman, Reduced development of COVID-19 in children reveals molecular checkpoints gating pathogenesis illuminating potential therapeutics. *Proc. Natl. Acad. Sci. U.S.A.* **117**, 24620–24626 (2020). doi:10.1073/pnas.2012358117 Medline
35. A. Kuri, B. M. Jacobs, N. Vickaryous, J. Pakpoor, J. Middeldorp, G. Giovannoni, R. Dobson, Epidemiology of Epstein-Barr virus infection and infectious mononucleosis in the United Kingdom. *BMC Public Health* **20**, 912 (2020). doi:10.1186/s12889-020-09049-x Medline
36. H.-X. Tan, W. S. Lee, K. M. Wragg, C. Nelson, R. Esterbauer, H. G. Kelly, T. Amarasena, R. Jones, G. Starkey, B. Z. Wang, O. Yoshino, T. Tiang, M. L. Grayson, H. Opdam, R. D'Costa, A. Vago, L. K. Mackay, C. L. Gordon, A. K. Wheatley, S. J. Kent, J. A. Juno; Austin Liver Transplant Perfusionist Group, Adaptive immunity to human coronaviruses is widespread but low in magnitude. *Clin. Transl. Immunology* **10**, e1264 (2021). doi:10.1002/cti2.1264 Medline
37. M. Koutsakos, P. T. Illing, T. H. O. Nguyen, N. A. Mifsud, J. C. Crawford, S. Rizzetto, A. A. Eltahla, E. B. Clemens, S. Sant, B. Y. Chua, C. Y. Wong, E. K. Allen, D. Teng, P. Dash, D. F. Boyd, L. Grzelak, W. Zeng, A. C. Hurt, I. Barr, S. Rockman, D. C. Jackson, T. C. Kotsimbos, A. C. Cheng, M. Richards, G. P. Westall, T. Loudovaris, S. I. Mannering, M. Elliott, S. G. Tangye, L. M. Wakim, J. Rossjohn, D. Vijaykrishna, F. Luciani, P. G. Thomas, S. Gras, A. W. Purcell, K. Kedzierska, Human CD8+ T cell cross-reactivity across influenza A, B and C viruses. *Nat. Immunol.* **20**, 613–625 (2019). doi:10.1038/s41590-019-0320-6 Medline
38. S. C. Clute, L. B. Watkin, M. Cornberg, Y. N. Naumov, J. L. Sullivan, K. Luzuriaga, R.

M. Welsh, L. K. Selin, Cross-reactive influenza virus-specific CD8+ T cells contribute to lymphoproliferation in Epstein-Barr virus-associated infectious mononucleosis. *J. Clin. Invest.* **115**, 3602–3612 (2005). doi:10.1172/JCI25078 Medline

39. H. Wedemeyer, E. Mizukoshi, A. R. Davis, J. R. Bennink, B. Rehermann, Cross-reactivity between hepatitis C virus and Influenza A virus determinant-specific cytotoxic T cells. *J. Virol.* **75**, 11392–11400 (2001). doi:10.1128/JVI.75.23.11392-11400.2001 Medline
40. S. G. Byars, S. C. Stearns, J. J. Boomsma, Association of Long-Term Risk of Respiratory, Allergic, and Infectious Diseases With Removal of Adenoids and Tonsils in Childhood. *JAMA Otolaryngol. Head Neck Surg.* **144**, 594–603 (2018). doi:10.1001/jamaoto.2018.0614 Medline
41. W. Sungnak, N. Huang, C. Bécavin, M. Berg, R. Queen, M. Litvinukova, C. Talavera-López, H. Maatz, D. Reichart, F. Sampaziotis, K. B. Worlock, M. Yoshida, J. L. Barnes; HCA Lung Biological Network, SARS-CoV-2 entry factors are highly expressed in nasal epithelial cells together with innate immune genes. *Nat. Med.* **26**, 681–687 (2020). doi:10.1038/s41591-020-0868-6 Medline
42. N. Huang, P. Pérez, T. Kato, Y. Mikami, K. Okuda, R. C. Gilmore, C. D. Conde, B. Gasmi, S. Stein, M. Beach, E. Pelayo, J. O. Maldonado, B. A. Lafont, S.-I. Jang, N. Nasir, R. J. Padilla, V. A. Murrah, R. Maile, W. Lovell, S. M. Wallet, N. M. Bowman, S. L. Meinig, M. C. Wolfgang, S. N. Choudhury, M. Novotny, B. D. Aevermann, R. H. Scheuermann, G. Cannon, C. W. Anderson, R. E. Lee, J. T. Marchesan, M. Bush, M. Freire, A. J. Kimple, D. L. Herr, J. Rabin, A. Grazioli, S. Das, B. N. French, T. Pranzatelli, J. A. Chiorini, D. E. Kleiner, S. Pittaluga, S. M. Hewitt, P. D. Burbelo, D. Chertow, K. Frank, J. Lee, R. C. Boucher, S. A. Teichmann, B. M. Warner, K. M. Byrd; NIH COVID-19 Autopsy Consortium; HCA Oral and Craniofacial Biological Network, SARS-CoV-2 infection of the oral cavity and saliva. *Nat. Med.* **27**, 892–903 (2021). doi:10.1038/s41591-021-01296-8 Medline
43. J. M. Schenkel, K. A. Fraser, L. K. Beura, K. E. Pauken, V. Vezys, D. Masopust, T cell memory. Resident memory CD8 T cells trigger protective innate and adaptive immune responses. *Science* **346**, 98–101 (2014). doi:10.1126/science.1254536 Medline
44. A. Chandran, J. Rosenheim, G. Nageswaran, L. Swadling, G. Pollara, R. K. Gupta, J. A. Guerra-Assuncao, A. Woolston, T. Ronel, C. Pade, J. Gibbons, B. S.-M. D. D. Estrada, M. R. de Massy, M. Whelan, A. Semper, T. Brooks, D. M. Altmann, R. J. Boyton, Á. McKnight, C. Manisty, T. A. Treibel, J. Moon, G. S. Tomlinson, M. K. Maini, B. M. Chain, M. Noursadeghi, Covid. Investigators, Non-severe SARS-CoV-2 infection is characterised by very early T cell proliferation independent of type 1 interferon responses and distinct from other acute respiratory viruses, *medRxiv*, 2021.03.30.21254540 (2021).
45. J. Zhao, J. Zhao, A. K. Mangalam, R. Channappanavar, C. Fett, D. K. Meyerholz, S. Agnihothram, R. S. Baric, C. S. David, S. Perlman, Airway Memory CD4(+) T Cells Mediate Protective Immunity against Emerging Respiratory Coronaviruses. *Immunity* **44**, 1379–1391 (2016). doi:10.1016/j.immuni.2016.05.006 Medline
46. J. S. Low, D. Vaqueirinho, F. Mele, M. Foglierini, J. Jerak, M. Perotti, D. Jarrossay, S. Jovic, L. Perez, R. Cacciatore, T. Terrot, A. F. Pellanda, M. Biggiogero, C. Garzoni, P. Ferrari, A. Ceschi, A. Lanzavecchia, F. Sallusto, A. Cassotta, Clonal analysis of immunodominance and cross-reactivity of the CD4 T cell response to SARS-CoV-2. *Science* **372**, 1336–1341 (2021). doi:10.1126/science.abg8985 Medline
47. L. Swadling, M. O. Diniz, N. M. Schmidt, O. E. Amin, A. Chandran, E. Shaw, C. Pade, J. M. Gibbons, N. L. Bert, A. T. Tan, A. Jeffery-Smith, C. Tan, C. Y. L. Tham, S. Kucyowicz, G. Aidoo-Micah, J. Rosenheim, J. Davies, M. P. Jensen, G. Joy, L. E. McCoy, A. M. Valdes, L. van Dorp, D. M. Altmann, R. J. Boyton, C. Manisty, T. A. Treibel, J. C. Moon, COVIDsortium investigators, Pre-existing polymerase-specific T cells expand in abortive seronegative SARS-CoV-2 infection, *medRxiv*, 2021.06.26.21259239 (2021).
48. N. G. Davies, P. Klepac, Y. Liu, K. Prem, M. Jit, R. M. Eggo; CMMID COVID-19 working group, Age-dependent effects in the transmission and control of COVID-19 epidemics. *Nat. Med.* **26**, 1205–1211 (2020). doi:10.1038/s41591-020-0962-9 Medline
49. G. Saletti, T. Gerlach, J. M. Jansen, A. Molle, H. Elbahesh, M. Ludlow, W. Li, B.-J. Bosch, A. D. M. E. Osterhaus, G. F. Rimmelzwaan, Older adults lack SARS CoV-2 cross-reactive T lymphocytes directed to human coronaviruses OC43 and NL63. *Sci. Rep.* **10**, 21447 (2020). doi:10.1038/s41598-020-78506-9 Medline
50. K. W. Ng, N. Faulkner, G. H. Cornish, A. Rosa, R. Harvey, S. Hussain, R. Ulferts, C. Earl, A. G. Wrobel, D. J. Benton, C. Roustan, W. Bolland, R. Thompson, A. Agua-Doce, P. Hobson, J. Heaney, H. Rickman, S. Paraskevopoulou, C. F. Houlihan, K. Thomson, E. Sanchez, G. Y. Shin, M. J. Spyer, D. Joshi, N. O'Reilly, P. A. Walker, S. Kjaer, A. Riddell, C. Moore, B. R. Jebson, M. Wilkinson, L. R. Marshall, E. C. Rosser, A. Radziszewska, H. Peckham, C. Ciurtin, L. R. Wedderburn, R. Beale, C. Swanton, S. Gandhi, B. Stockinger, J. McCauley, S. J. Gamblin, L. E. McCoy, P. Cherepanov, E. Nastouli, G. Kassiotis, Preexisting and de novo humoral immunity to SARS-CoV-2 in humans. *Science* **370**, 1339–1343 (2020). doi:10.1126/science.abe1107 Medline
51. B. Slütter, N. Van Braeckel-Budimir, G. Abboud, S. M. Varga, S. Salek-Ardakani, J. T. Harty, Dynamics of influenza-induced lung-resident memory T cells underlie waning heterosubtypic immunity. *Sci. Immunol.* **2**, eaag2031 (2017). doi:10.1126/sciimmunol.aag2031 Medline
52. N. Van Braeckel-Budimir, S. M. Varga, V. P. Badovinac, J. T. Harty, Repeated Antigen Exposure Extends the Durability of Influenza-Specific Lung-Resident Memory CD8^{+} T Cells and Heterosubtypic Immunity. *Cell Rep.* **24**, 3374–3382.e3 (2018). doi:10.1016/j.celrep.2018.08.073 Medline
53. J. Theorell-Haglöw, C. B. Miller, D. J. Bartlett, B. J. Yee, H. D. Openshaw, R. R. Grunstein, Gender differences in obstructive sleep apnoea, insomnia and restless legs syndrome in adults - What do we know? A clinical update. *Sleep Med. Rev.* **38**, 28–38 (2018). doi:10.1016/j.smrv.2017.03.003 Medline
54. K. Katoh, K. Misawa, K. Kuma, T. Miyata, MAFFT: A novel method for rapid multiple sequence alignment based on fast Fourier transform. *Nucleic Acids Res.* **30**, 3059–3066 (2002). doi:10.1093/nar/gkf436 Medline
55. P. J. A. Cock, T. Antao, J. T. Chang, B. A. Chapman, C. J. Cox, A. Dalke, I. Friedberg, T. Hamelryck, F. Kauff, B. Wilczynski, M. J. L. de Hoon, Biopython: Freely available Python tools for computational molecular biology and bioinformatics. *Bioinformatics* **25**, 1422–1423 (2009). doi:10.1093/bioinformatics/btp163 Medline
56. D. A. Price, J. M. Brenchley, L. E. Ruff, M. R. Betts, B. J. Hill, M. Roederer, R. A. Koup, S. A. Migueles, E. Gostick, L. Wooldridge, A. K. Sewell, M. Connors, D. C. Douek, Avidity for antigen shapes clonal dominance in CD8+ T cell populations specific for persistent DNA viruses. *J. Exp. Med.* **202**, 1349–1361 (2005). doi:10.1084/jem.20051357 Medline
57. J. Niessl, A. E. Baxter, A. Morou, E. Brunet-Ratnasingham, G. Sannier, G. Gendron-Lepage, J. Richard, G.-G. Delgado, N. Brassard, I. Turcotte, R. Fromentin, N. F. Bernard, N. Chomont, J.-P. Routy, M. Dubé, A. Finzi, D. E. Kaufmann, Persistent expansion and Th1-like skewing of HIV-specific circulating T follicular helper cells during antiretroviral therapy. *EBioMedicine* **54**, 102727 (2020). doi:10.1016/j.ebiom.2020.102727 Medline
58. C. Klumpp-Thomas, H. Kalish, M. Drew, S. Hunsberger, K. Snead, M. P. Fay, J. Mehalko, A. Shunmugavel, V. Wall, P. Frank, J.-P. Denson, M. Hong, G. Gulten, S. Messing, J. Hicks, S. Michael, W. Gillette, M. D. Hall, M. J. Memoli, D. Esposito, K. Sadtler, Standardization of ELISA protocols for serosurveys of the SARS-CoV-2 pandemic using clinical and at-home blood sampling. *Nat. Commun.* **12**, 113 (2021). doi:10.1038/s41467-020-20383-x Medline

Acknowledgments: We express our gratitude to all donors, health care personnel, study coordinators, administrators, and laboratory managers involved in this work. **FUNDING:** J.N. was supported by an EMBO Postdoctoral Fellowship (ALTF 1062-2020). J.L. was supported by a Wenner-Gren Postdoctoral Fellowship. D.A.P. was supported by the UK Coronavirus Immunology Consortium funded by the National Institute for Health Research/UK Research and Innovation. D.F. was supported by the Acta Oto-Laryngologica Stiftelse. M.B. was supported by the Swedish Research Council, the Karolinska Institutet, the Jeansson Stiftelser, the Åke Wibergs Stiftelse, the Swedish Society of Medicine, the Swedish Cancer Society, the Hedlunds Stiftelse, the Lars Hiertas Stiftelse, the Jonas Söderquist Stiftelse, and the Clas Groschinskys Minnesfond. **AUTHOR CONTRIBUTIONS:** Conceptualization: J.N., T.S., and M.B.; investigation: J.N., T.S., and J.L.; formal analysis: J.N.; visualization: J.N.; resources: V.K., M.F., J.M., A.R., L.M., E.K., W.W., S.L.-L., E.B.H., A.C.K., J.F., J.S., D.A.P., J.M., and D.F.; funding acquisition: D.A.P., J.M., D.F., and M.B.; supervision: D.A.P. and M.B.; writing – original draft: J.N. and M.B.; writing – review and editing: D.A.P. and M.B. **COMPETING INTERESTS:** M.B. is a consultant for Oxford Immunotec. **DATA AND MATERIALS AVAILABILITY:** HLA class I tetramers can be generated and shared

on a collaborative basis. Raw data for all graphs are included in Table S5. All data needed to evaluate the conclusions in the paper are present in the paper or the Supplementary Materials. This work is licensed under a Creative Commons Attribution 4.0 International (CC BY 4.0) license, which permits unrestricted use, distribution, and reproduction in any medium, provided the original work is properly cited. To view a copy of this license, visit https://creativecommons.org/licenses/by/4.0/. This license does not apply to figures/photos/artwork or other content included in the article that is credited to a third party; obtain authorization from the rights holder before using such material.

Submitted 22 June 2021
Accepted 3 September 2021
Published First Release 14 September 2021
10.1126/sciimmunol.abk0894

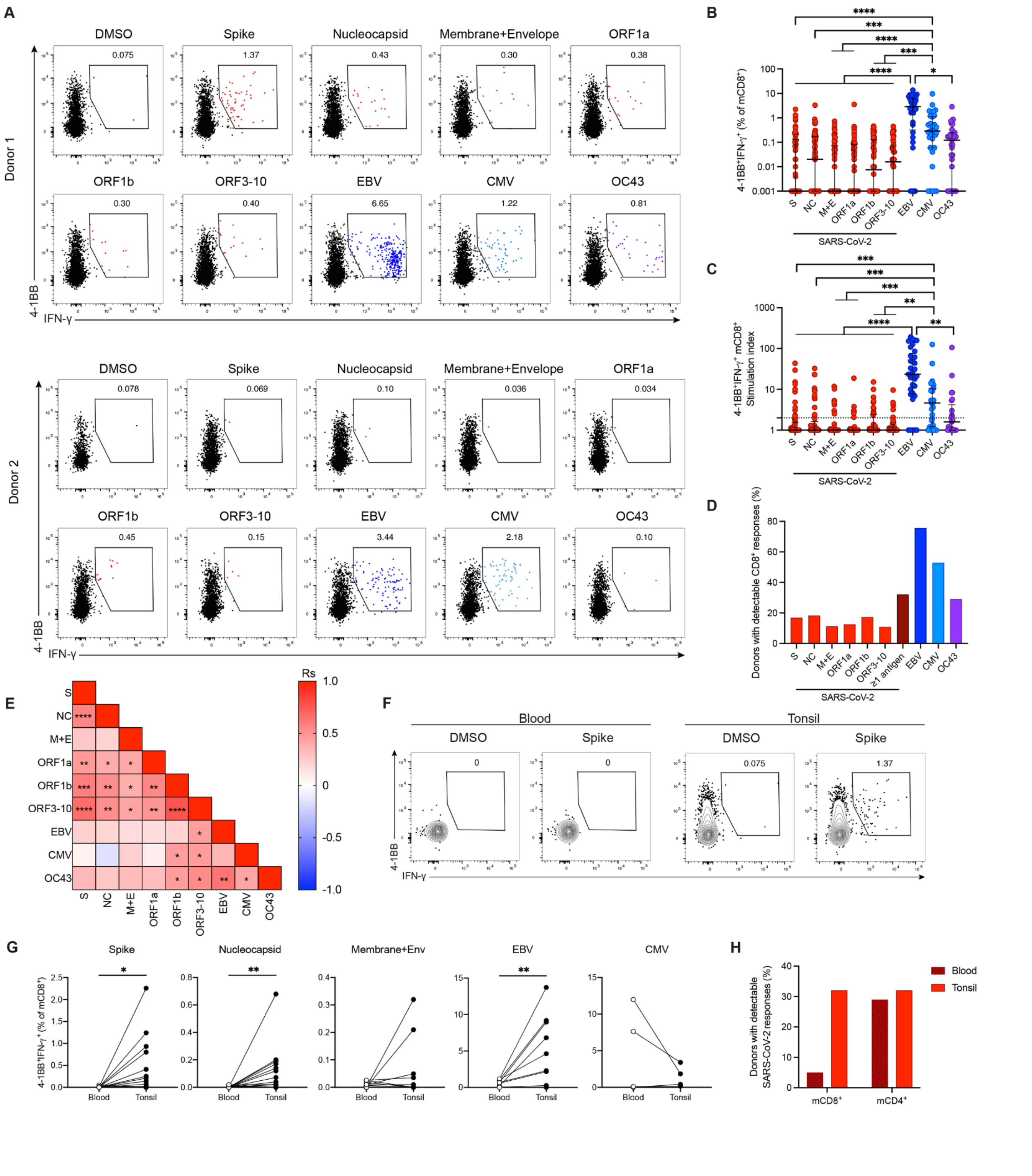
A
DMSO
Spike
Nucleocapsid
Membrane+Envelope
ORF1a
ORF1b
ORF3-10
EBV
CMV
OC43
Donor 1
Donor 2
4-1BB
IFN-γ
B
C
D
SARS-CoV-2
E
Rs
F
Blood
Tonsil
G
H
Blood
Tonsil
mCD8+
mCD4+

Fig. 1. Identification of tonsillar mCD8$^+$ T cells specific for SARS-CoV-2 in unexposed individuals. Tonsil cells from children (n = 40) and adults (n = 41) were stimulated with overlapping peptide pools and analyzed for intracellular expression of 4-1BB/IFN-γ. (**A**) Representative plots showing the gating of 4-1BB$^+$IFN-γ$^+$ mCD8$^+$ T cells from individuals with responses to several SARS-CoV-2 proteins (top) or one SARS-CoV-2 protein (bottom). (**B**) Net frequencies (background subtracted) of 4-1BB$^+$IFN-γ$^+$ mCD8$^+$ T cells. (**C**) Stimulation indices for 4-1BB$^+$IFN-γ$^+$ mCD8$^+$ T cells calculated as fold change relative to the negative control. The dotted line indicates a stimulation index of 2. (**D**) Frequency of tonsil samples with positive antigen-specific mCD8$^+$ T cell responses (stimulation index ≥2). (**E**) Correlation matrix showing associations between stimulation indices for virus-specific mCD8$^+$ T cells. (**F**) Representative gating of 4-1BB$^+$IFN-γ$^+$ mCD8$^+$ T cells in matched blood and tonsil samples. (**G**) Frequencies of 4-1BB$^+$IFN-γ$^+$ mCD8$^+$ T cells in matched blood and tonsil samples. (**H**) Frequency of blood or tonsil samples with positive mCD4$^+$ or mCD8$^+$ T cell responses to SARS-CoV-2. (B–E) S: spike; NC: nucleocapsid; M+E: membrane+envelope. (B, C) Graphs show median ± IQR. Kruskal-Wallis test with Dunn's post-test. (E) Spearman correlations. (G) Wilcoxon signed-rank test. **P* < 0.05, **P < 0.01, ****P* < 0.001, *****P* < 0.0001.

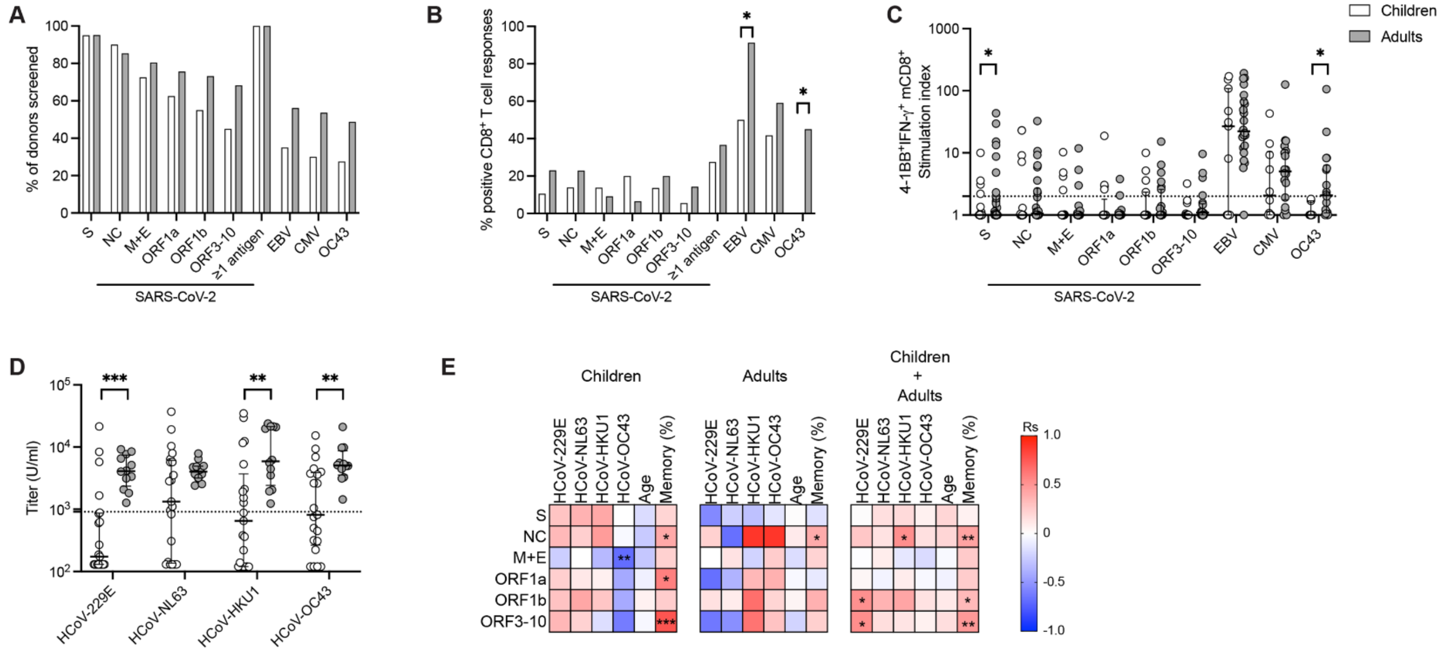

Fig. 2. Magnitude and prevalence of tonsillar mCD8⁺ T cells specific for SARS-CoV-2 in unexposed children and adults. Virus-specific tonsillar mCD8⁺ T cell responses were compared between children (white bars/symbols; n = 40) and adults (grey bars/symbols; n = 41). (**A**) Overall frequency of tonsil samples tested with individual peptide pools. (**B**) Overall frequency of tonsil samples with positive mCD8⁺ T cell responses (stimulation index ≥2) to different antigens. (**C**) Stimulation indices for 4-1BB⁺IFN-γ⁺ mCD8⁺ T cells calculated as fold change relative to the negative control. The dotted line indicates a stimulation index of 2. (**D**) Antibody titers (U/ml) for HCoVs in children (n = 21) and adults (n = 12). The dotted line represents a seropositivity threshold calculated on the basis of SARS-CoV-2 spike trimer-specific antibody titers as shown in Fig. S5A. (**E**) Correlation matrices showing associations between stimulation indices for SARS-CoV-2-specific mCD8⁺ T cell responses, HCoV antibody titers, age, and total frequencies of mCD8⁺ T cells for children, adults, or both cohorts. (A–C) S: spike; NC: nucleocapsid; M+E: membrane+envelope. (B) Fisher's exact test. (C, D) Graphs show median ± IQR. Mann-Whitney test. (E) Spearman correlation. *$P < 0.05$, **$P < 0.01$, ***$P < 0.001$.

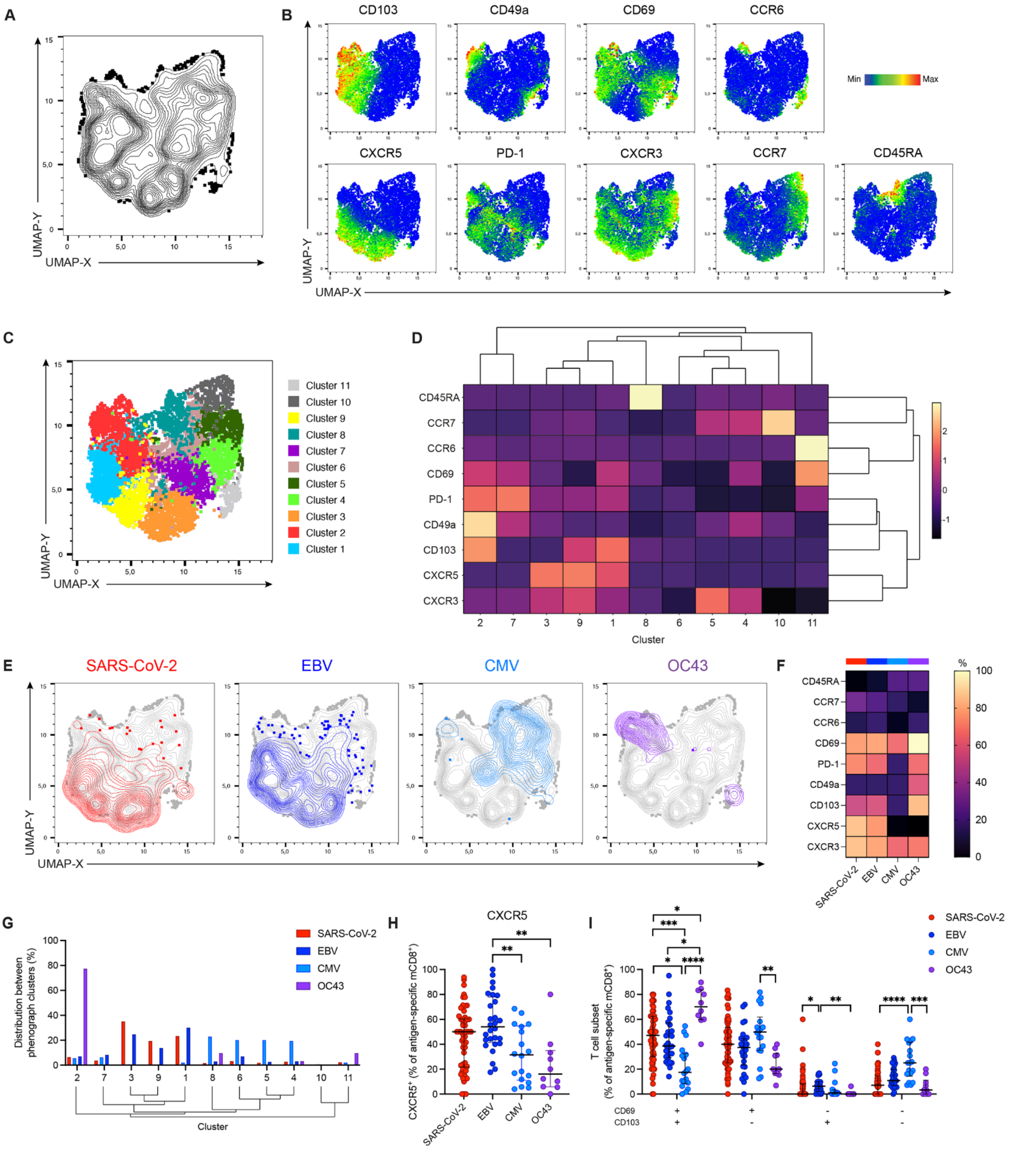
A
B
CD103
CD49a
CD69
CCR6
Min
Max
CXCR5
PD-1
CXCR3
CCR7
CD45RA
UMAP-Y
UMAP-X
C
D
Cluster 11
Cluster 10
Cluster 9
Cluster 8
Cluster 7
Cluster 6
Cluster 5
Cluster 4
Cluster 3
Cluster 2
Cluster 1
Cluster
E
SARS-CoV-2
EBV
CMV
OC43
F
G
Distribution between phenograph clusters (%)
H
CXCR5
CXCR5+ (% of antigen-specific mCD8+)
I
T cell subset (% of antigen-specific mCD8+)
CD69
CD103

Fig. 3. Tonsillar SARS-CoV-2-specific mCD8⁺ T cells in unexposed individuals express CD103, CD69, and CXCR5. (**A**) UMAP representation showing the clustering of concatenated total and virus-specific mCD8⁺ T cells from representative individuals (n = 4). (**B**) Expression of individual surface markers overlaid on the UMAP plot derived from (A). (**C**) UMAP representation derived from (A) with subpopulations (n = 11) identified using Phenograph. (**D**) Hierarchical clustering of expression intensity for the indicated markers in each subpopulation identified in (C). (**E**) Distribution of virus-specific mCD8⁺ T cells on the UMAP plot derived in (A). (**F**) Median percent expression of the indicated markers among virus-specific mCD8⁺ T cells. (**G**) Distribution of virus-specific mCD8⁺ T cells among clusters derived using Phenograph. Clusters are hierarchical as indicated in (D). (**H**) Frequency of CXCR5⁺ virus-specific mCD8⁺ T cells. (**I**) Comparison of virus-specific mCD8⁺ subsets based on the expression of CD69/CD103. (H, I) Graphs show median ± IQR. Kruskal-Wallis test with Dunn's post-test. *$P < 0.05$, **$P < 0.01$, ***$P < 0.001$, ****$P < 0.0001$.

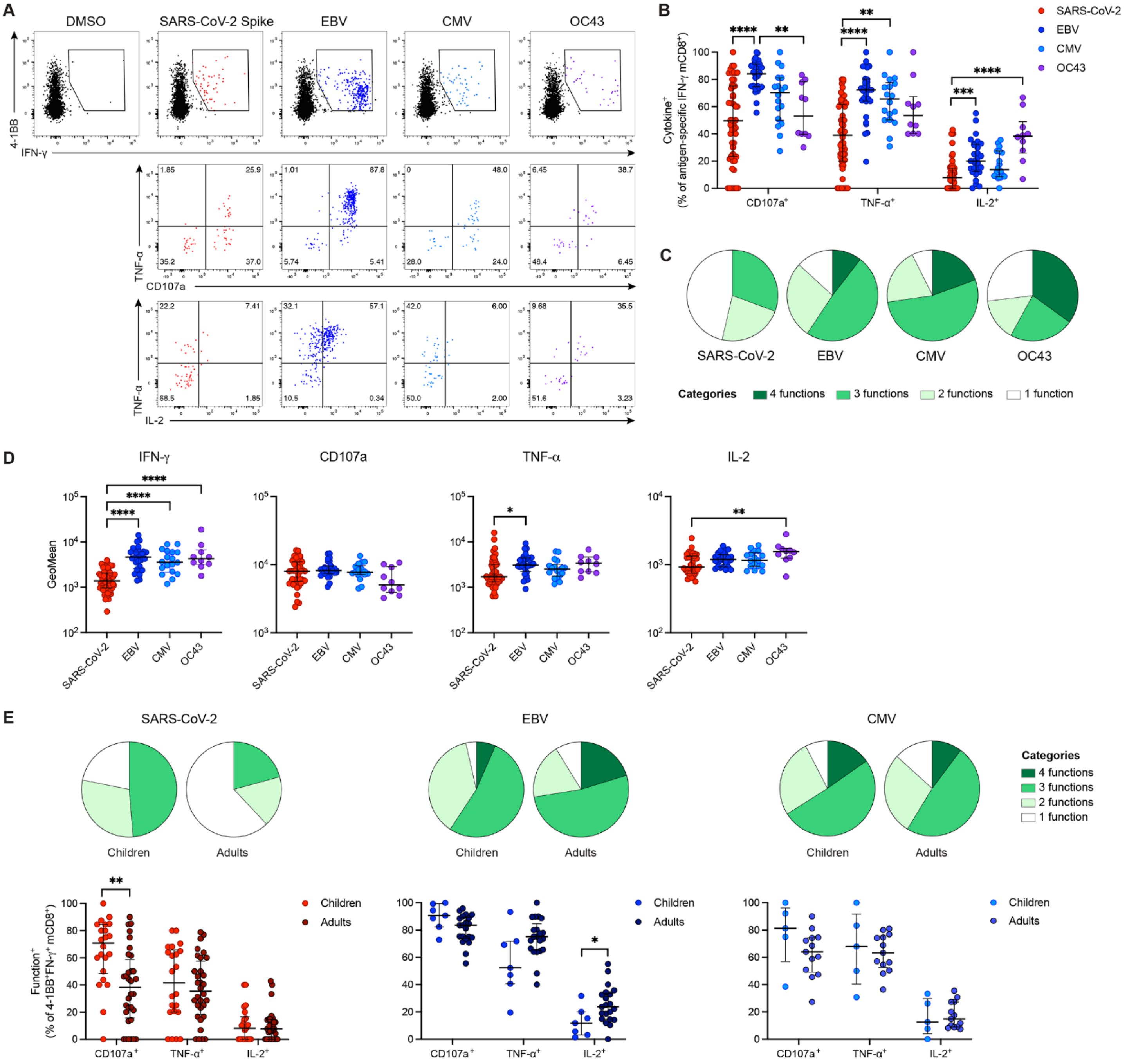

Fig. 4. Functional profiles of virus-specific tonsillar mCD8+ T cells. (**A**) Representative plots showing coexpression of CD107a/TNF-α or IL-2/TNF-α among 4-1BB+IFN-γ+ virus-specific mCD8+ T cells. (**B**) Frequencies of CD107a+, TNF-α+, or IL-2+ virus-specific mCD8+ T cells. (**C**) Pie chart summarizing the functional profiles of virus-specific mCD8+ T cells. (**D**) Geometric mean (GeoMean) fluorescence values for IFN-γ, CD107a, TNF-α, and IL-2 among function+ virus-specific mCD8+ T cells. (**E**) Functional profiles and frequencies of CD107a+, TNF-α+, or IL-2+ virus-specific mCD8+ T cells in children and adults. Graphs show median (C, E) or median ± IQR (B, D, E). (B, D) Kruskal-Wallis test with Dunn's post-test. (E) Mann-Whitney test. *$P < 0.05$, **$P < 0.01$, ***$P < 0.001$, ****$P < 0.0001$
