# Supplementary material for: Identification of resident memory CD8+ T cells with functional specificity for SARS-CoV-2 in unexposed oropharyngeal lymphoid tissue
Source: Sci Immunol. 2021 Oct 22;6(64):eabk0894. doi: 10.1126/sciimmunol.abk0894 (PMC10763663; doi:10.1126/sciimmunol.abk0894)
Supplement: Supplementary file 2 — Figs. S1 to S7 Tables S1 to S4 [file sciimmunol.abk0894_sm.pdf]

Science Immunology
AAAS

Supplementary Materials for

Identification of resident memory $CD8^+$ T cells with functional specificity for SARS-CoV-2 in unexposed oropharyngeal lymphoid tissue

Julia Niessl *et al.*

Corresponding author: Julia Niessl, julia.niessl@ki.se; Marcus Buggert, marcus.buggert@ki.se

Sci. Immunol. **6**, eabk0894 (2021)
DOI: 10.1126/sciimmunol.abk0894

The PDF file includes:

Figs. S1 to S7
Tables S1 to S4

Other Supplementary Material for this manuscript includes the following:

Table S5

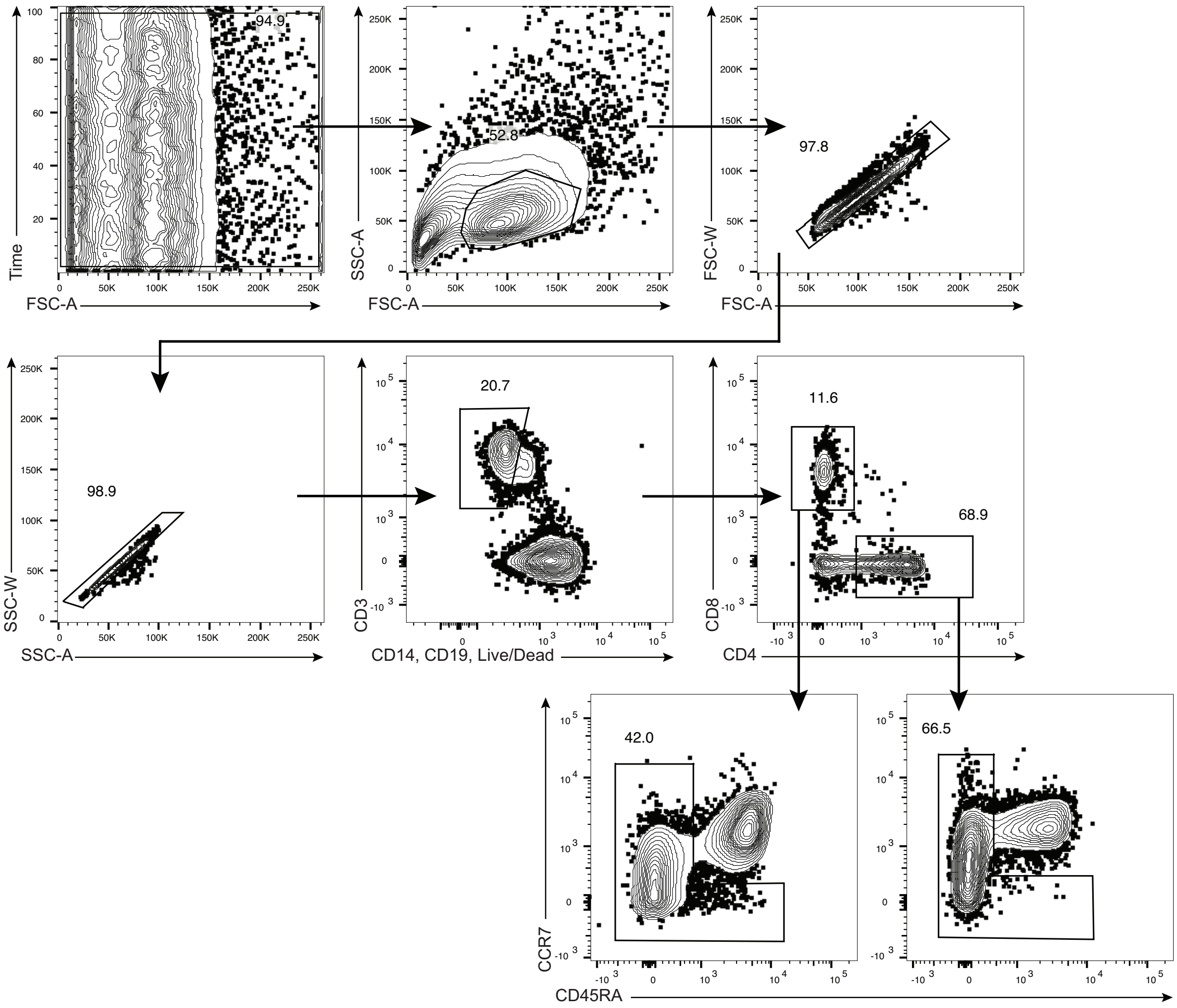

Figure S1. Flow cytometric gating strategy for the identification of mCD4$^+$ and mCD8$^+$ T cells.

Numbers indicate percentages in the drawn gates.

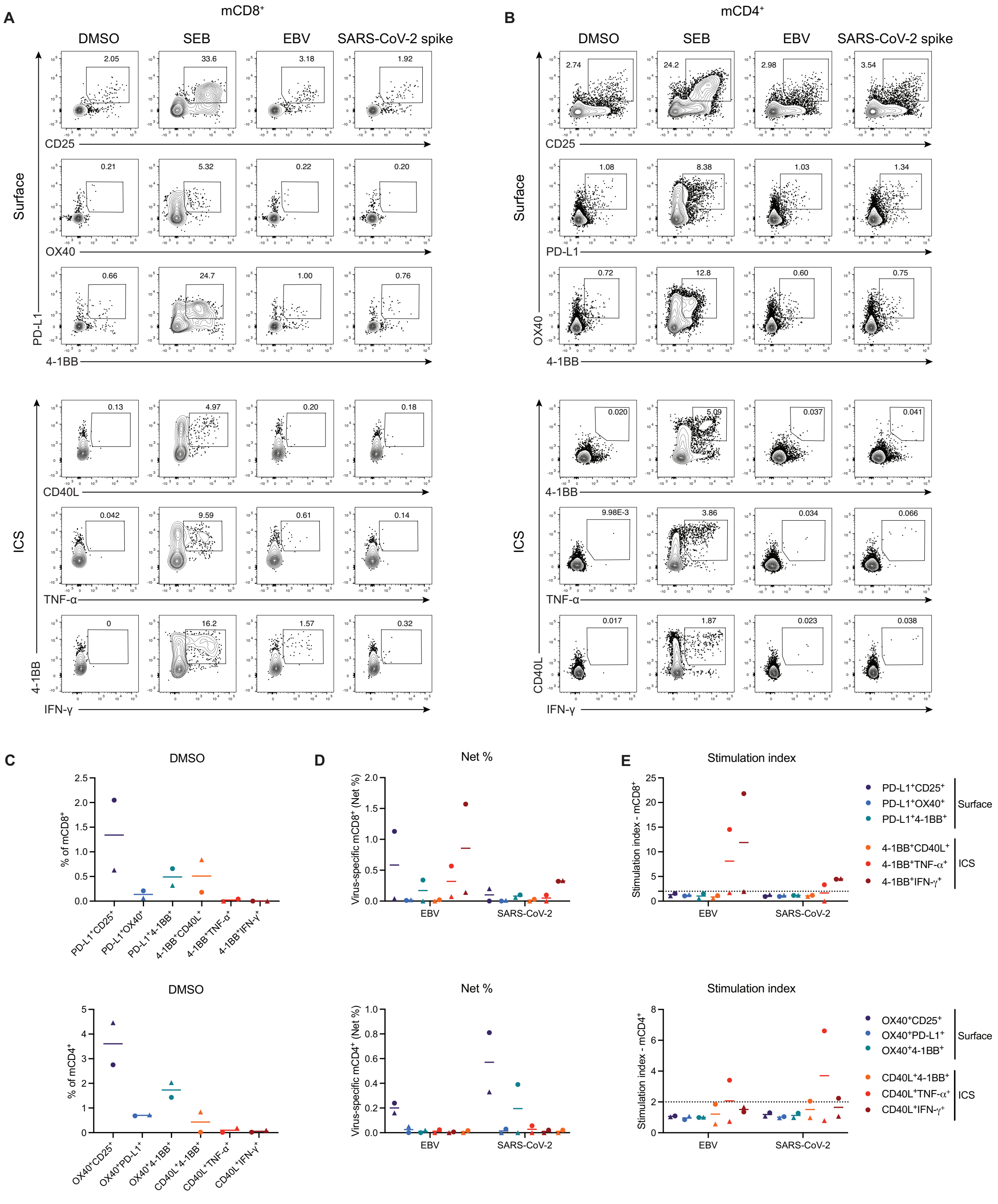

Figure S2. Assessment of activation-induced markers and cytokines for the detection of virus-specific tonsillar mCD4⁺ and mCD8⁺ T cells.

Tonsil cells were stimulated with the indicated peptides or SEB and analyzed for surface expression of activation-induced markers (AIMs) or intracellular expression of AIMs and cytokines (ICS). Negative control wells contained equivalent DMSO. (**A**, **B**) Representative

plots showing the expression profile of surface AIMs (top) and intracellular AIMs/cytokines (bottom) for tonsillar mCD8$^+$ (A) or mCD4$^+$ T cells (B). (**C**) Frequencies of mCD8$^+$ (top) or mCD4$^+$ T cells (bottom) expressing the indicated markers in the absence of stimulation. (**D**) Net frequencies (background subtracted) of virus-specific mCD8$^+$ (top) or mCD4$^+$ T cells (bottom) identified by different marker combinations. (**E**) Stimulation indices for virus-specific mCD8$^+$ (top) or mCD4$^+$ T cells (bottom) calculated as fold change relative to the negative control. The dotted line indicates a stimulation index of 2. (C to E) Symbols represent different individuals (n = 2).

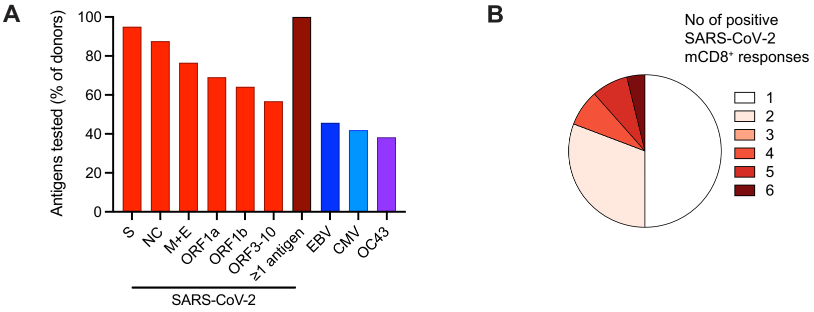

Figure S3. Evaluation of SARS-CoV-2-specific mCD8$^+$ T cell responses

(**A**) Overall frequency of tonsil samples tested with individual peptide pools. n = 81. (**B**) Overall number of antigens recognized in the same tonsil sample pooled for all donors with positive mCD8$^+$ T cell responses against at least one antigen derived from SARS-CoV-2. n = 26 (only tonsil samples, for which all 6 SARS-CoV-2 antigens were tested, are included).

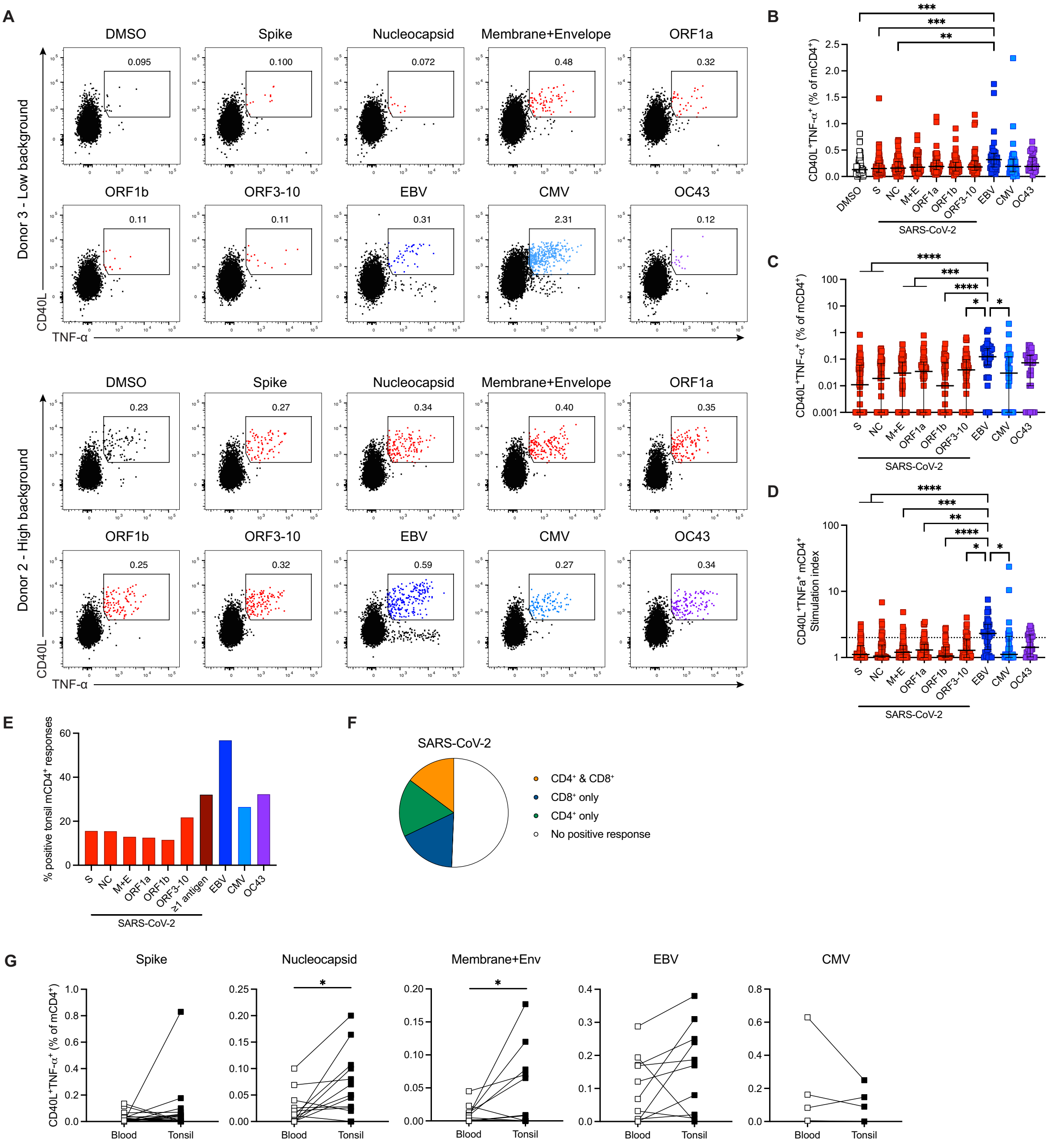

Figure S4. Identification of tonsillar mCD4$^+$ T cells specific for SARS-CoV-2 in unexposed individuals.

Tonsil cells were stimulated with the indicated peptides and analyzed for intracellular coexpression of CD40L and TNF-α. (**A**) Representative plots showing the gating of CD40L$^+$TNF-α$^+$ mCD4$^+$ T cells from individuals with low (top) or high background levels of activation (bottom). (**B**) Frequencies of CD40L$^+$TNF-α$^+$ mCD4$^+$ T cells. (**C**) Net frequencies (background subtracted) of CD40L$^+$TNF-α$^+$ mCD4$^+$ T cells. (**D**) Stimulation indices for

CD40L$^+$TNF-α^+ mCD4$^+$ T cells calculated as fold change relative to the negative control. The dotted line indicates a stimulation index of 2. (**E**) Overall frequency of tonsil samples with positive mCD4$^+$ T cell responses (stimulation index ≥2) to the indicated antigens. (**F**) Overall frequency of tonsil samples with positive mCD4$^+$ and/or mCD8$^+$ T cell responses (stimulation index ≥2) to SARS-CoV-2. (**G**) Frequencies of CD40L$^+$TNF-α^+ mCD4$^+$ T cells in matched blood and tonsil samples. (B–E) S, spike; NC, nucleocapsid; M+E, membrane+envelope. (B–D) Graphs show median ± IQR. Kruskal-Wallis test with Dunn's posttest. (G) Wilcoxon signed rank test. *$P < 0.05$; **$P < 0.01$; ***$P < 0.001$; ****$P < 0.0001$.

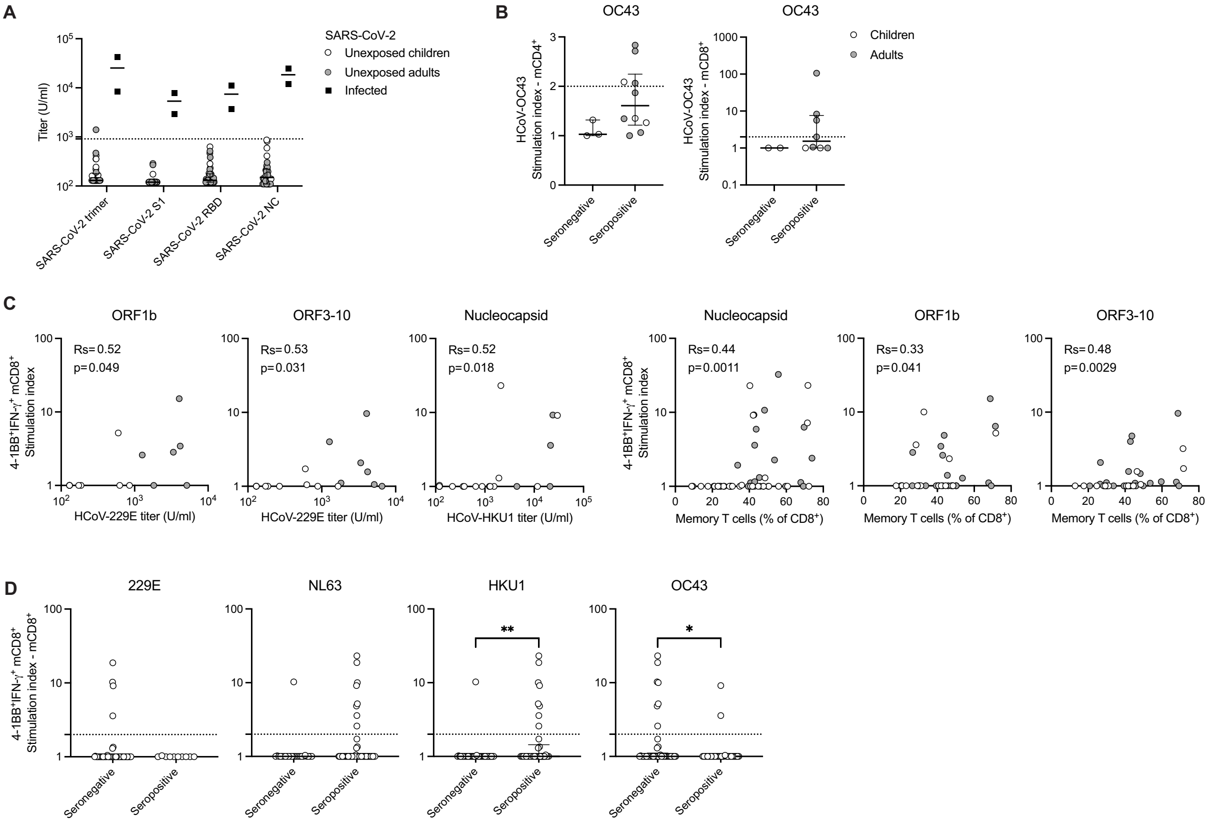

Figure S5. Evaluation of SARS-CoV-2-specific mCD8$^+$ T cell responses and HCoV antibody titers in children and adults.

(**A**) Titers for different SARS-CoV-2-spike-specific antibodies measured by ProcartaPlex assay in unexposed children (n=21), adults (n=12) or SARS-CoV-2 convalescent individuals (n=2). Dotted line represents threshold of seropositivity calculated based on SARS-CoV-2 spike trimer-specific antibody values in unexposed individuals (mean + (3 * SD)). This threshold was applied to Ig measurements shown in Fig. 2D. (**B**) Stimulation indices for HCoV-OC43-specific mCD4$^+$ (left) and mCD8$^+$ (right) T cell responses in HCoV-OC43 seronegative or seropositive individuals. Dotted line represents stimulation index of 2. **(C**) Associations between 4-1BB$^+$IFN-γ^+ mCD8$^+$ T cell stimulation indices for the indicated SARS-CoV-2 antigens and indicated HCoV antibody titers (U/ml) or total mCD8$^+$ T cell frequencies with corresponding Spearman R and *P* values. (**D**) Stimulation indices for all SARS-CoV-2-specific mCD8$^+$ T cell responses in indicated HCoV-seronegative or -seropositive children. Dotted line represents stimulation index of 2. (A) S, spike; NC, nucleocapsid; RBD, receptor binding

domain. (A, B, D) Graphs show median ± IQR. (C) Spearman correlations. (D) Mann-Whitney test. *P < 0.05; **P < 0.01.

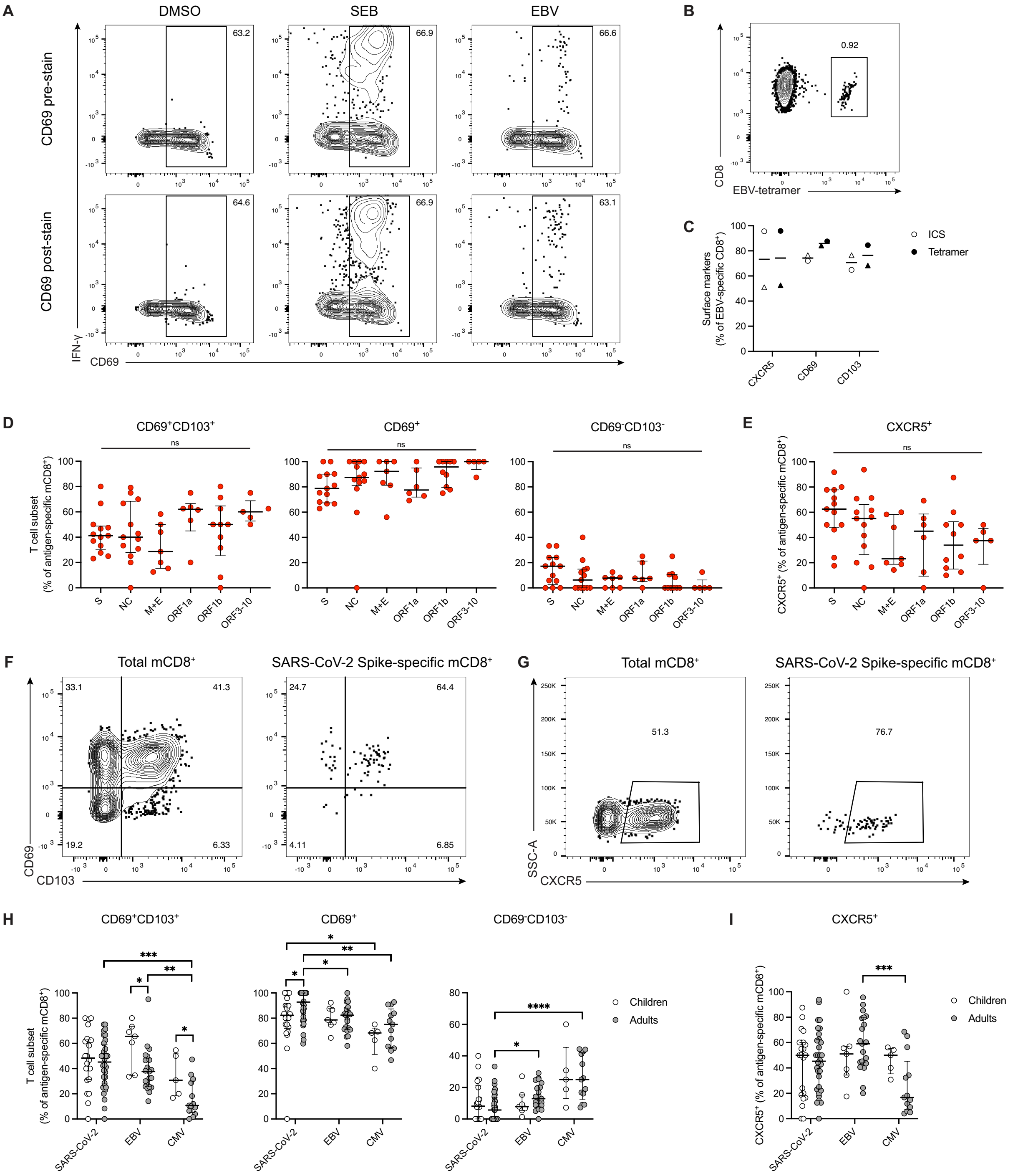

Figure S6. Comparability and stability of the tonsillar mCD8⁺ T cell phenotype.

(**A**) Representative plots showing intracellular expression of IFN-γ and surface expression of CD69 among mCD8⁺ T cells. Surface staining for CD69 was performed either before (top) or after stimulation (bottom). (**B**) Representative plot showing EBV tetramer staining of tonsillar mCD8⁺ T cells from one individual. (**C**) Frequencies of CXCR5⁺, CD69⁺, or CD103⁺ EBV-specific mCD8⁺ T cells identified as tetramer⁺ or 4-1BB⁺IFN-γ⁺ (ICS). Symbols represent

different individuals (n=2). (**D**, **E**) Expression profiles for CD69/CD103 (D) or CXCR5 (E) among SARS-CoV-2-reactive $mCD8^+$ T cells specific for the indicated antigens. (**F**, **G**) Representative plots showing expression of CD69/CD103 (F) or CXCR5 (G) among total (left) or SARS-CoV-2 spike-specific $mCD8^+$ T cells (right) from one individual. (**H**, **I**) Expression profiles for CD69/CD103 (H) or CXCR5 (I) among virus-specific $mCD8^+$ T cells from children or adults. (D, E) S, spike; NC, nucleocapsid; M+E, membrane+envelope. (C) Graphs show median. (D, E, H, I) Graphs show median ± IQR. (D, E) Kruskal-Wallis test with Dunn's post-test. (H, I) Mann-Whitney test for comparisons between children and adults, Kruskal-Wallis test with Dunn's post-test for comparisons between antigens. $^*P < 0.05$; $^{**}P < 0.01$; $^{***}P < 0.001$; $^{****}P < 0.0001$; ns, not significant.

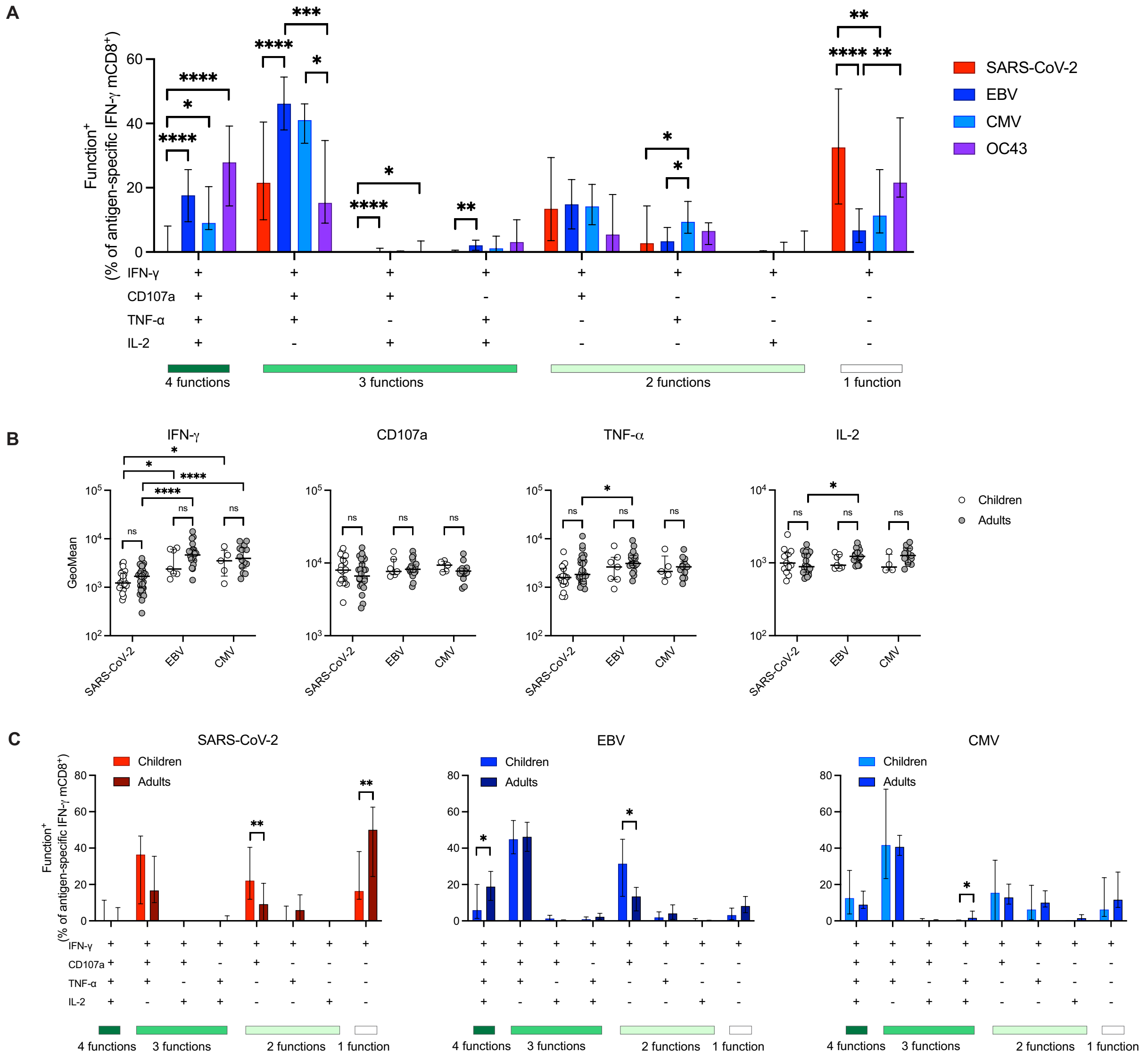

Figure S7. Functional profiles of virus-specific tonsillar mCD8$^+$ T cells.

(**A**) Coexpression of CD107a, TNF-α, and IL-2 among 4-1BB$^+$IFN-γ^+ virus-specific mCD8$^+$ T cells. (**B**) Geometric mean (GeoMean) fluorescence values for the same functions in children and adults. (**C**) Coexpression of CD107a, TNF-α, and IL-2 among 4-1BB$^+$IFN-γ^+ virus-specific mCD8$^+$ T cells from children or adults. (A-C) Graphs show show median ± IQR. (A) Kruskal-Wallis test with Dunn's post-test. (B) Kruskal-Wallis test with Dunn's post-test for comparisons among virus specificities within each age group; Mann-Whitney test for comparisons between children and adults within virus specificities. (C) Mann-Whitney test. *P < 0.05; **P < 0.01; ***P < 0.001; ****P < 0.0001; ns, not significant.

Table S1. Donor characteristics.

Tonsil cohorts, SARS-CoV-2 unexposed	*Children (n = 40)*	*Adults (n = 41)*
Age (median [range], years)	3 [2–5]	41 [28–67]
Sex (male, female)	23, 17	37, 4
Year of surgery	2015–2016	2016–2018

SARS-CoV-2 convalescent	*n = 2*
Age (years)	32, 35
Sex (male, female)	2, 0
Year of collection	2021

Table S2. Seroprevalence for HCoVs in children and adults

	Seropositive children		*Seropositive adults*	
	Number	*Percentage*	*Number*	*Percentage*
HCoV-229E	4/21	19.1	12/12	100
HCoV-NL63	13/21	62.0	12/12	100
HCoV-HKU1	9/21	42.9	12/12	100
HCoV-OC43	10/21	47.6	12/12	100
Any HCoV	18/21	85.7	12/12	100

Table S3. Associations between mCD8$^+$ T cell responses specific for SARS-CoV-2 and mCD8$^+$ T cell responses specific for EBV, CMV, or HCoV-OC43.

Children		*Neg*	*Pos*	*Total*	*P **
EBV					0.0047
	Neg	7	0	7	
	Pos	1	6	7	
CMV					0.2222
	Neg	6	1	7	
	Pos	2	3	5	
OC43					NA
	Neg	6	5	11	
	Pos	0	0	0	

Adults		Neg	Pos	Total	*P **
EBV					0.4862
	Neg	2	0	2	
	Pos	11	10	21	
CMV					0.4149
	Neg	6	3	9	
	Pos	6	7	13	
OC43					0.1984
	Neg	8	4	12	
	Pos	3	6	9	

*Fisher’s exact test. NA, not applicable.

Table S4. Flow cytometry reagents and analysis software.

	Reagent	Catalog number	Source	RRID
Antibodies	Anti-CCR4─BB700 (clone 1G1)	566475	BD Biosciences	RRID:AB_2744302
	Anti-CCR6─BUV737 (clone 11A9)	612780	BD Biosciences	RRID:AB_2870109
	Anti-CCR7─APC-Cy7 (clone G043H7)	353212	BioLegend	RRID:AB_10916390
	Anti-CD3─BUV805 (clone UCHT1)	612895	BD Biosciences	RRID:AB_2870183
	Anti-CD3─BV605 (clone UCHT1)	300460	BioLegend	RRID:AB_2564380
	Anti-CD4─BUV496 (clone SK3)	612936	BD Biosciences	RRID:AB_2870220
	Anti-CD4─PE-Cy5.5 (clone S3.5)	MHCD0418	Thermo Fisher Scientific	RRID:AB_10376013
	Anti-CD8─BUV395 (clone RPA-T8)	563795	BD Biosciences	RRID:AB_2722501
	Anti-CD14─BV510 (clone M5E2)	301842	BioLegend	RRID:AB_2561946
	Anti-CD19─BV510 (clone HIB19)	302242	BioLegend	RRID:AB_2561668
	Anti-CD45RA─BV570 (clone HI100)	304132	BioLegend	RRID:AB_2563813
	Anti-CD49a─BUV615 (clone SR84)	751437	BD Biosciences	RRID:AB_2875433
	Anti-CD69─BUV563 (clone FN50)	748764	BD Biosciences	RRID:AB_2873167
	Anti-CD103─BV605 (clone Ber-ACT8)	350218	BioLegend	RRID:AB_2564283
	Anti-CD107a─BV785 (clone H4A3)	328644	BioLegend	RRID:AB_2565967
	Anti-CD134─PE (clone Ber-ACT35)	350004	BioLegend	RRID:AB_10645478
	Anti-CD137─PE-Cy7 (clone 4B4-1)	309818	BioLegend	RRID:AB_2207741
	Anti-CD154─BV421 (clone 24-31)	310824	BioLegend	RRID:AB_2562721
	Anti-CXCR3─BV750 (clone 1C6)	746895	BD Biosciences	RRID:AB_2871692
	Anti-CXCR5─BB515 (clone RF8B2)	564624	BD Biosciences	RRID:AB_2738871
	Anti-IFN-γ─FITC (clone B27)	554700	BD Biosciences	RRID:AB_395517
	Anti-IFN-γ─PE (clone B27)	506507	BD Biosciences	RRID:AB_315440
	Anti-IL-2─PE-Dazzle594 (clone MQ1-17H12)	500344	BioLegend	RRID:AB_2564091
	Anti-IL-17A─eFluor660 (clone eBio64CAP17)	50-7178-42	Thermo Fisher Scientific	RRID:AB_2574282
	Anti-PD-1─BV711 (clone EH12.2H7)	329928	BioLegend	RRID:AB_2562911
	Anti-PD-L1─BV786 (clone 29E.2A3)	329735	BioLegend	RRID:AB_2629581
	Anti-TNF-α─BV650 (clone Mab11)	502936	BD Biosciences	RRID:AB_2563884
Other reagents	BD GolgiStop	554724	BD Biosciences	-
	Brefeldin A	420601	BioLegend	-
	Brilliant Stain Buffer Plus	566385	BD Biosciences	RRID:AB_2869761
	Dasatinib	73082	STEMCELL	-
	DNase I	4716728001	Sigma-Aldrich	-
	FoxP3 Transcription Factor Staining Buffer Set	00-5523-00	Thermo Fisher Scientific	-
	LIVE/DEAD Fixable Aqua Dead Cell Stain Kit	L34957	Thermo Fisher Scientific	-
	Paraformaldehyde	22023	Biotium	-
	Staphylococcal enterotoxin B (SEB)	S4881	Sigma-Aldrich	-
Software	FlowJo	-	FlowJo, LLC	RRID:SCR_008520
	Prism	-	GraphPad Software Inc.	RRID:SCR_002798
	Bioplex Manager	-	Biorad	RRID:SCR_014330
